# Comparison of dengue case classification schemes and evaluation of biological changes in different dengue clinical patterns in a longitudinal follow-up of hospitalized children in Cambodia

**DOI:** 10.1371/journal.pntd.0008603

**Published:** 2020-09-14

**Authors:** Philippe Dussart, Veasna Duong, Kevin Bleakley, Camille Fortas, Patrich Lorn Try, Kim Srorn Kim, Rithy Choeung, Saraden In, Anne-Claire Andries, Tineke Cantaert, Marie Flamand, Philippe Buchy, Anavaj Sakuntabhai

**Affiliations:** 1 Virology Unit, Institut Pasteur du Cambodge, Institut Pasteur International Network, Phnom Penh, Cambodia; 2 Laboratoire de mathématiques d'Orsay, Université Paris-Saclay, CNRS, Inria, Orsay, France; 3 Epidemiology and Public Health Unit, Institut Pasteur du Cambodge, Institut Pasteur International Network, Phnom Penh, Cambodia; 4 Pediatric Department, Kampong Cham Provincial hospital, Kampong Cham, Cambodia; 5 Immunology Unit, Institut Pasteur du Cambodge, Institut Pasteur International Network, Phnom Penh, Cambodia; 6 Structural Virology Unit, Department of Virology, Institut Pasteur, CNRS UMR 3569, Paris, France; 7 GlaxoSmithKline Vaccines R&D, Singapore; 8 Functional Genetics of Infectious Diseases Unit, Department of Genomes and Genetics, Institut Pasteur, Paris, France; 9 Centre National de la Recherche Scientifique, Génomique évolutive, modélisation et santé, UMR 2000, Paris, France; Institute for Disease Modeling, UNITED STATES

## Abstract

**Background:**

The World Health Organization (WHO) proposed guidelines on dengue clinical classification in 1997 and more recently in 2009 for the clinical management of patients. The WHO 1997 classification defines three categories of dengue infection according to severity: dengue fever (DF), dengue hemorrhagic fever (DHF), and dengue shock syndrome (DSS). Alternative WHO 2009 guidelines provide a cross-sectional classification aiming to discriminate dengue fever from dengue with warning signs (DWWS) and severe dengue (SD). The primary objective of this study was to perform a comparison of two dengue classifications. The secondary objective was to describe the changes of hematological and biochemical parameters occurring in patients presenting with different degrees of severity during the course of the disease, since progression to more severe clinical forms is unpredictable.

**Methodology/Principal findings:**

We performed a prospective, monocentric, cross-sectional study of hospitalized children in Cambodia, aged from 2 to 15 years old with severe and non-severe dengue. We enrolled 243 patients with acute dengue-like illness: 71.2% were dengue infections confirmed using quantitative reverse transcription PCR or NS1 antigen capture ELISA, of which 87.2% and 9.0% of DF cases were respectively classified DWWS and SD, and 35.9% of DHF were designated SD using an adapted WHO 2009 classification for SD case definition. Systematic use of ultrasound at patient admission was crucial for detecting plasma leakage. No difference was observed in the concentration of secreted NS1 protein between different dengue severity groups. Lipid profiles were different between DWWS and SD at admission, characterized by a decrease in total cholesterol, HDL cholesterol, and LDL cholesterol, in SD.

**Conclusions/Significance:**

Our results show discrepancies between the two classifications, including misclassification of severe dengue cases as mild cases by the WHO 1997 classification. Using an adapted WHO 2009 classification, SD more precisely defines the group of patients requiring careful clinical care at a given time during hospitalization.

## Author summary

Dengue is a viral disease that results in various health conditions, ranging from strictly asymptomatic infections to life-threatening severe dengue. Here we have compared the two World Health Organization (WHO) dengue classification schemes from 1997 and 2009, which define different categories of dengue fever and have been at the root of much debate in the dengue research community. We enrolled a Cambodian pediatric cohort of hospitalized dengue-confirmed patients, with clinical and biological follow-up. Our findings demonstrate that (i) the WHO 1997 classification is a longitudinal dengue-case classification which uses strict prerequisite clinical and/or biological signs to move from one level of severity to another, and (ii) the WHO 2009 classification, being a transversal dengue-case classifier, is more flexible and when adapted with systematic use of ultrasound is more accurate in identifying severe dengue case for timely and appropriate clinical management of hospitalized patients. Our laboratory results found that lipid profiles exhibited changes and differences between different dengue severity groups at hospital.

## Introduction

Dengue is a viral disease transmitted by *Aedes aegypti* and *Aedes albopictus* mosquitoes. The global affected population is estimated at 390 million individuals across more than 100 countries, with about 70% of infections occurring in Asia [1]. This disease, formerly limited to tropical or sub-tropical areas, is now reaching new regions with temperate or Mediterranean climates [2–5]. Dengue virus (DENV) belongs to the genus *Flavivirus* and comprises four distinct serotypes: DENV-1, DENV-2, DENV-3, and DENV-4 [6]. Infection by one of the four serotypes results in short-term heterotypic immunity. Moreover, although reinfections with the same serotype have been reported [7], serotype-specific immunity is usually considered to be lifelong. Progression to more serious disease is frequently, but not exclusively, associated with secondary infection by heterologous serotypes [8]. The clinical characteristics of dengue are wide-ranging, from mild conditions to life-threatening symptoms. More rarely, atypical occurrences of dengue virus infection may involve fulminant hepatitis, cardiomyopathy, acute renal failure, and encephalopathy [9–12]. The course of the disease can be divided into three phases: (i) acute febrile phase lasting 2 to 7 days with non-specific clinical signs and possible mild hemorrhagic symptoms (petechial and mucosal bleeding), (ii) critical phase generally occurring at the time of defervescence, during which complications may appear in a small proportion of patients, including severe hemorrhage, plasma leakage with shock, and organ impairment, and (iii) recovery phase. Early and appropriate management of dengue cases with severe symptoms is a key step in reducing mortality [6].

The World Health Organization (WHO) proposed the first guidelines on dengue with a clinical classification in 1974, later revised in 1997 [13,14]. This WHO 1997 classification scheme, which comprises three categories–dengue fever (DF), dengue hemorrhagic fever (DHF), and dengue shock syndrome (DSS)–is based on prerequisite clinical and/or biological signs to correctly classify the degree of severity of the disease [15,16]. However, as dengue case rates increased and the disease became widespread in Asia and the Americas, many clinicians confronted with the full spectrum of dengue encountered difficulties in applying WHO 1997 case definitions for triage and clinical management [15–18]. Subsequent prospective clinical studies aimed to improve dengue case management [19,20], and the WHO launched new case management guidelines in 2009 (WHO 2009), which instead of being longitudinal (previous step required before passing to next step), provided a cross-sectional classification of dengue cases that established criteria to identify dengue cases with warning signs (DWWS) or severe dengue (SD) [21]. Deciding which of these significantly different classification schemes (WHO 1997 or WHO 2009) clinicians should rely on has been a stumbling block in dengue fever research ever since. Several studies using different methods and datasets to compare the two schemes have been published [19,20,22–26].

Thus, the primary objective of the current study is to perform a comparison of the two dengue classification schemes–using an adapted WHO 2009 classification for SD case definition–in a cross-sectional cohort of Cambodian children aged from 2 to 15 years old, hospitalized in Kampong Cham province hospital with confirmed dengue, and benefiting from clinical and biological follow-ups. The secondary objective is to describe in detail the changes of hematological and biochemical parameters–including lipid profiles–occurring in children presenting with different degrees of dengue disease severity at hospital admittance and during the course of their illness.

## Methods

### Ethics statement

The study was approved by the Cambodian National Ethics Committee for Human Research (approval #087NECHR/2011). All patient inclusion and blood sampling occurred after obtaining written informed consent from the patient’s parents or guardians.

### Study participants

Patients presenting with acute dengue-like illness during two consecutive dengue epidemic seasons–between June and October of 2011 and 2012 –were enrolled at Kampong Cham Referral Hospital. Inclusion criteria, following the WHO 1997 classification scheme, were: children between 2 and 15 years old who had fever or history of fever at presentation and onset within previous days of at least two of the following symptoms: headache, retro-orbital pain, muscle pain, joint pain, rash, or any bleeding signs. Exclusion criteria were symptoms inconsistent with dengue, obvious non-dengue acute infections (e.g., otitis media, pneumonia, meningitis), or a known chronic illness.

### Clinical methods

We performed a prospective, monocentric, cross-sectional study of hospitalized children with severe and non-severe dengue. The first visit was conducted at admission (Visit 1). The day of onset of symptoms was defined as day 0 of the illness. Visit 2 was conducted at the defervescence phase, which is characterized as the first day with temperature ≤ 38 °C in the absence of antipyretic intake. Finally, Visit 3 was performed and considered the pre-discharge/discharge visit for patients who recovered entirely, or a follow-up visit for patients in the critical phase. A clinical and biological follow-up was conducted at each visit as described below.

### Blood collection and laboratory analyses

For all included patients, whole blood was collected in two tubes with EDTA anticoagulant shortly after admission (Visit 1), at defervescence (Visit 2), and at discharge (Visit 3). The volume drawn was adapted according to participant weight: 3.0 mL for children below 20 kg, and 5.0 mL for those over 20 kg [27]. One tube from each visit was processed by the laboratory at Kampong Cham Hospital for initial blood tests. The other tube and the remaining blood from the first tube were sent with ice packs to the Institut Pasteur du Cambodge (in Phnom Penh) within 5–10 hours for complementary analyses including lipid profiles (triglycerides, total cholesterol, HDL cholesterol, and calculated LDL cholesterol), C-reactive protein (CRP), total and conjugated bilirubin, aspartate aminotransferase (AST) and alanine aminotransferase (ALT), and dengue diagnosis as described below. The total cholesterol, HDL cholesterol and triglycerides levels were measured using the COBAS INTEGRA 400 plus analyzer (Roche Diagnostics. Meylan, France) as part of a clinical laboratory routine. We did not measure the LDL cholesterol level directly in our study cohort, but instead used the calculation method previously defined by Freidwald et al. based on total cholesterol, HDL cholesterol, and triglyceride levels [28].

In order to assess a patient's clinical and biological evolution towards either a possible recovery phase or critical phase–characterized mainly by an increase of capillary permeability in parallel with increasing hematocrit level [21]–patients were followed during their hospitalization until having 2 days without fever, with daily clinical examination and serial hematocrit and platelet counts recorded. Defervescence was characterized as having no fever for 48 hours. In the meantime, ultrasonographic examinations of the abdomen and chest were also systematically performed at admission, defervescence, and discharge to identify either a recovery phase or any evidence of mild or severe plasma leakage possibly for evidence of a critical phase or severe dengue.

### Classification of arbovirus infections and non-dengue infections

DENV infection of hospitalized patients was confirmed by NS1 antigen detection using the capture ELISA assay described by Andries et al. [29] and/or by RT-qPCR and/or virus isolation on *Aedes albopictus* C6/36 cells on the plasma sample obtained at Visit 1 [29,30] and/or DENV-specific IgM seroconversion and/or a fourfold increase in the titer of total antibodies using the HI assay in paired acute and convalescent sera [21] (S1 Table). Considering that Cambodia is highly endemic for DENV and Japanese encephalitis virus (JEV) [31,32], and due to potential cross-reactivity among flaviviruses and the presence of other arboviruses, all sera collected during the acute and convalescent phases of infection, respectively Visits 1 and 3, were tested for anti-DENV, anti-JEV, and anti-Chikungunya virus (CHIKV), using an in-house IgM capture ELISA (MAC-ELISA) and hemagglutination inhibition (HI) assay as previously described [33,34]. Dengue immune status, distinguishing between primary and secondary infection, was determined using WHO guidelines [14]: primary infections were characterized by the presence or absence of HI antibodies in acute-phase samples and by low titers with a 4-fold rise of HI antibodies (≤ 1:1,280) in serum from the convalescence phase with an elapsed time of at least 7 days between admission (Visit 1) and discharge samples (Visit 3). Conversely, secondary infections were defined by the presence of HI antibodies in acute-phase samples and by high titers with a 4-fold rise of HI antibodies (≥ 1:2,560) in serum from the convalescence phase. However, when the elapsed time between the admission and discharge samples was not optimal; i.e., less than 7 days, all admission samples collected within the first three days after symptoms onset with negative HI titers associated to discharged samples with HI titers ≤ 1,280 were considered as primary infections.

Other arbovirus infections were defined as follows: acute Chikungunya infections were diagnosed by positive RT-PCR [35] or CHIV-specific IgM seroconversion [36]; acute dengue and Chikungunya coinfection was diagnosed by positive RT-PCR for both viruses; recent infection was characterized by detection of IgM antibodies against DENV, JEV or flavivirus (detection of both anti-DENV and anti-JEV antibodies) and/or CHIKV (S1 Table). Patients were classified as non-dengue when laboratory tests showed no presence of virus RNA by RT-qPCR or NS1 antigen by capture ELISA, and no presence or a fourfold increase of DENV-specific antibodies by MAC-ELISA or HI assay, respectively (S1 Table). Finally, the severity of the disease among confirmed dengue patients was assessed according to the WHO 1997 and WHO 2009 criteria using clinical, biological and ultrasound data recorded at admission, defervescence and discharge [14,21].

### WHO 1997 and 2009 classifications

The severity of the disease among confirmed dengue patients was assessed according to the WHO 1997 and WHO 2009 criteria using clinical, biological and ultrasound data recorded at admission, defervescence and discharge. As per the WHO 1997 case definition, patients were classed into three groups: dengue fever (DF), dengue hemorrhagic fever (DHF), and dengue shock syndrome (DSS) [14]. In addition, patients were classified according to WHO 2009 classification criteria, separating dengue cases into severe dengue and non-severe dengue. Non-severe dengue patients were divided into two subgroups: patients with warning signs (DWWS) and those without (D-nonWS). DWWS was defined as an association of clinical signs with laboratory findings. Moreover, clinical fluid accumulation (pleural effusion and ascites fluid) and liver enlargement were evaluated using ultrasonographic examination. We adapted WHO 2009 classification by using ultrasound that gave us the opportunity to semi-quantify the degree of fluid accumulation, providing a more accurate clinical diagnosis in discriminating between DWWS and severe dengue (SD): (i) minimal amount of liquid was considered as mild plasma leakage i.e. a warning sign (DWWS); (ii) moderate or abundant amount of liquid was considered as severe dengue, possibly leading to aggravation with respiratory distress (SD). Both classifications were applied after the final assessment using all available biological and clinical information. See S1 Appendix for a detailed description of the classification criteria.

### Statistical analysis

Statistical analyses were performed evaluate differences between patient subgroups in terms of demographic, biological and clinical characteristics using GraphPad Prism for Windows, version 8.0.1 (GraphPad Software, Inc., La Jolla, CA, USA) and the statistical software R [37].

First, acute dengue infection (DI, n = 173), other arbovirus infection (OAI, n = 33), and non-dengue infection (NDI, n = 37) subgroups were compared. Fisher exact tests were used to test categorical variables, and one-way ANOVA (or non-parametric Kruskall-Wallis tests for non-Gaussian data) to test continuous variables. If the one-way ANOVA or Kruskall-Wallis test was significant (p<0.01), post-hoc Tukey tests were used to investigate which pairwise differences were significant.

Second, differences in demographics (gender and age), dengue immune status, laboratory evidence of dengue infection, and hospitalization duration of acute dengue-infected children included in the study across the subgroups of both the WHO 1997 and adapted WHO 2009 dengue classification schemes (n = 173), were tested for statistical significance. Fisher exact tests were used to test categorical variables and one-way ANOVA or Kruskall-Wallis tests for continuous variables.

Third, a comparison of laboratory findings was conducted between dengue case groups using the WHO 1997 and adapted WHO 2009 dengue case definitions. Depending on the variable being examined, t-tests, Mann-Whitney tests, or one-way ANOVA (followed by Tukey's test if necessary) were used to evaluate statistically significant differences. No corrections for multiple testing were made, but a more stringent p-value cut-off was used for to consider significance (p<0.01) in this article.

## Results

### Characteristics of included patients

Between June and October of 2011 and 2012, 243 hospitalized patients with acute dengue-like illness were enrolled in the study, including 109 males and 134 females (male to female ratio: 0.82; mean age ± SD: 8.2 ± 3.3 years; median: 8 years). Among the enrolled patients, 173 (71.2%) were laboratory confirmed acute dengue infections (DI), 33 (13.6%) were diagnosed as other arbovirus infections (OAI), and dengue infection was not found in 37 (15.2%) patients (Non-dengue infection: NDI). Demographic, clinical, and biological characteristics of included patients at hospital admission are in presented in Table 1.

**Table 1:** Demographic, clinical and biological characteristics of included patients at hospital admission (n = 243).

	Acute dengue infection (n = 173)		Other arbovirus infection (n = 33)		Non-dengue infection (n = 37)		p-value*
	n	[95% CI] or (%)	n	[95%] CI or (%)	n	[95% CI] or (%)	
**Demographic characteristics and medical history**							
Mean age (years) (±SD; median) (years)	7.7	[7.3–8.2] (± 3.0; 7.0)	9.1	[7.8–10.4] (± 3.7; 8.0)	9.8	[8.7–10.9] (± 3.3; 10.0)	**4.1e-4**
Sex ratio (male:female)	0.82		0.83		0.76		0.98
Day after onset of symptoms	3.6	[3.3–3.8]	2.8	[2.3–3.3]	2.5	[1.9–3.0]	**4.2e-5**
**Clinical examination**							
Weight (kg)	19.3	[18.2–20.4]	23.7	[19.7–27.7]	23.3	[20.5–26.2]	**1.3e-3**
Height (cm)	115	[112.5–117.5]	125	[117.5–132.6]	124.3	[118–130.5]	**7.6e-4**
Temperature (°C)	37.8	[37.7–38.0]	38.1	[37.6–38.6]	38.25	[37.9–38.6]	0.07
Systolic blood pressure (mmHg)	99.1	[97.4–100.8]	101.8	[97–106.6]	102.6	[98.4–106.8]	0.17
Diastolic blood pressure (mmHg)	64.3	[62.5–66.1]	68.9	[63.1–74.7]	63.6	[60.1–67.2]	0.11
**Symptoms at admission**							
Headache	152	(88)	31	(94)	31	(84)	0.47
Retro-orbital pain	117	(68)	22	(67)	25	(68)	1.00
Muscle pain	88	(51)	18	(55)	15	(41)	0.45
Joint pain	40	(23)	8	(24)	10	(27)	0.89
Backache	10	(6)	1	(3)	3	(8)	0.75
**Hemorrhagic manifestations**							
Petechiae	37	(21)	3	(9)	3	(8)	0.07
Ecchymosis or purpura	21	(12)	3	(9)	0	-	0.06
Bleeding nose, gums	7	(4)	1	(3)	1	(3)	1.00
Conjunctival hemorrhage	37	(21)	3	(9)	8	(22)	0.25
Hematemesis	3	(2)	3	(9)	0	-	0.06
Tourniquet test	121	(70)	21	(64)	19	(51)	0.09
At least one manifestation	136	(79)	23	(70)	25	(68)	0.24
**Warning signs**							
Vomiting	139	(80)	26	(79)	24	(65)	0.12
Abdominal pain	126	(73)	25	(76)	22	(60)	0.22
Diarrhea	7	(4)	0	-	0	-	0.57
Hepatomegaly (by echography only)	74	(43)	18	(56)	8	(22)	**9.2e-3**
At least one warning sign	162	(94)	30	(91)	30	(81)	0.05
**Plasma leakage**							
Ascites	71	(41)	16	(50)	6	(16)	**4.5e-3**
Pleural effusion	66	(38)	13	(41)	4	(11)	**2.3e-3**
Facial oedema	10	(6)	0	-	1	(3)	0.49
At least one plasma leakage sign	83	(48)	17	(52)	8	(22)	**8.1e-3**
**Shock signs**							
Cold extremities	28	(16)	6	(18)	1	(3)	0.05
Weak pulse	16	(9)	5	(15)	0	-	0.05
Narrow pulse pressure	31	(18)	9	(27)	2	(6)	0.05
Cyanosis	21	(13)	5	(15)	0	-	0.08
Delayed capillary refill time	26	(15)	5	(15)	1	(3)	0.20
At least one shock sign	38	(22)	10	(30)	2	(5)	0.02
**Other complications**							
Coma or convulsions	3	(2)	1	(3)	0	-	0.52
Other	6	(5)	0	-	1	(8)	0.39
**Baseline laboratory findings**							
*** Hematological parameters***							
White blood cell count (10^9^/L)	5.4	[4.8–5.9]	6.9	[5.5–8.4]	8.9	[7.5–10.4]	**1.6e-6**
Red blood cell count (10^12^/L)	5	[4.8–5.1]	4.8	[4.5–5.0]	4.7	[4.6–4.9]	0.07
Hemoglobin (g/L)	12.2	[12–12.5]	11.9	[11.2–12.6]	11.5	[11.2–11.9]	0.07
Hematocrit (%)	38.5	[37.8–39.2]	37	[34.8–39.1]	36.7	[35.6–37.7]	0.03
Platelet count (10^9^/L)	103	[91–115]	116	[86–147]	232	[200–263]	**4.9e-15**
*** Liver function***							
Aspartate Aminotransferase (IU/L)	190	[160–220]	148	[82–214]	56	[37–75]	**5.1e-11**
Alanine Aminotransferase (IU/L)	76	[64–88]	64	[30–98]	34	[20–47]	**1.3e-6**
Total Bilirubin (μmol/L)	4.4	[4.0–4.8]	4.8	[4.0–5.6]	5.3	[3.6–7.1]	0.22
Conjugated Bilirubin (μmol/L)	1.5	[1.3–1.7]	1.6	[1.2–2.0]	1.7	[1.1–2.3]	0.74
*** Inflammation***							
C-Reactive Protein (mg/L)	10.3	[6.5–14.2]	14.8	[8.5–21.2]	25.9	[15.8–36]	**1.4e-4**
*** Lipid profile***							
Total cholesterol (mmol/L)	2.9	[2.8–3.0]	3	[2.7–3.3]	3.4	[3.1–3.7]	**1.1e-3**
HDL cholesterol (mmol/L)	0.6	[0.5–0.6]	0.6	[0.5–0.7]	0.8	[0.7–1.0]	**3.4e-4**
LDL cholesterol (mmol/L)	1.4	[1.3–1.5]	1.6	[1.3–1.9]	2.0	[1.8–2.3]	**9.1e-5**
Triglycerides (mmol/L)	1.9	[1.8–2.1]	1.8	[1.4–2.2]	1.2	[1.0–1.5]	**8.4e-4**

* A Fisher exact test was used for categorical variables, and one-way ANOVA or Kruskall-Wallis test for continuous variables. If the one-way ANOVA or Kruskall-Wallis test was significant (p<0.01), post-hoc Tukey tests were used to investigate which pairwise differences were significant: *Age*: acute dengue infection (DI) vs. non-dengue infection (NDI), p = 8.6e-4. *Day after onset of symptoms*: DI vs. NDI, p = 1.6e-4; *Height*: DI vs. NDI, p = 0.01; DI vs. OAI, p = 8.2e-3. *White blood cell count*: DI vs. NDI, p = 1.5e-6. *Platelet count*: DI vs. NDI, p = 9.7e-15; NDI vs. OAI, p = 6.3e-8. *AST*: DI vs. NDI, p = 3.3e-4. *ALT*: DI vs. NDI, p = 0.01. *CRP*: DI vs. NDI, p = 2.9e-3. *Total cholesterol*: DI vs. NDI, p = 6.8e-4. *HDL cholesterol*: DI vs. NDI, p = 2.6e-4; NDI vs. OAI, p = 5.3e-3. *LDL cholesterol*: DI vs. NDI, p = 6e-5. *Triglycerides*: DI vs. NDI, p = 5e-4. All other pairwise differences were not statistically significant.

#### Demographic parameters

The mean age in the DI group (7.7 years) was significantly different (younger) than that in the NDI group (9.8 years) (Tukey's test: p = 8.6e-4). Moreover, the average number of days after onset of symptoms for arrival at hospital was significantly higher for DI (3.6 days) than NDI (2.5 days) (Tukey's test: p = 1.6e-4 –Table 1).

#### Clinical parameters

Clinical and physical examinations at hospital admission found that the proportions of patients with hepatomegaly and/or plasma leakage (ascites and pleural effusion) were significantly different across the three conditions (DI, OAI, NDI); hepatomegaly: (43%, 56%, 22%), p = 9.2e-3; ascites: (41%, 50%, 16%), p = 4.5e-3; pleural effusion: (38%, 41%, 11%), p = 2.3e-3. Note also that the presence of at least one plasma leakage sign at admission was associated with significantly different proportions of patients across the three conditions (Fisher exact test, p = 8.1e-3). On the other hand, hemorrhagic manifestations and other warning signs were not different across the three groups at Visit 1. Interestingly, the OAI group, which most likely includes a few subjects for whom the diagnosis of dengue fever could not be formally proven, includes subjects (n = 10, 30.3%) with at least one sign of shock (Table 1).

#### Biological parameters

Hematological and biochemical findings of included patients at hospital admission are also summarized in Table 1. Of note, lipid profile results at Visit 1 showed that total cholesterol, HDL cholesterol and LDL cholesterol all had significantly lower means in the DI group than in the NDI group, while triglyceride had a significantly higher mean in the DI group than in the NDI group.

### Laboratory-confirmed dengue infections

Among the 173 laboratory-confirmed dengue cases, DENV-1 was the predominant serotype (n = 162, 93.6%) followed by DENV-2 (n = 2, 1.2%). The DENV serotype was not conclusive in 5 NS1 antigen positive patients (2.9%) and 4 additional DENV-specific IgM seroconverted patients (2.3%–Table 2 and S1 Table). Dengue immune status testing indicated that 127 (73.4%) patients were undergoing a secondary immune response, 29 (16.8%) a primary immune response, and 17 (9.8%) were undetermined (Table 2). The average number of days after onset of symptoms at hospital arrival was significantly higher for DSS (4.6 days) than DHF (3.8 days) (t-test: p = 1.3e-3) and for SD (4.0 days) than DWWS (3.3 days) (t-test: p = 4.6e-3,–Table 2). No difference in hospitalization length was observed between the different clinical forms of DI, regardless of the WHO classification used.

**Table 2:** Demographic (gender and age), dengue immune status, laboratory-confirmed dengue diagnosis and hospitalization duration of acute dengue-infected children included in the study in terms of the WHO 1997 and adapted WHO 2009 dengue classifications (n = 173).

		Acute dengue infection															
		All acute dengue cases (n = 173)		WHO 1997							WHO 2009						
				DF (n = 78)		DHF (n = 78)		DSS (n = 17)		p-value	D-nonWS (n = 3)		DWWS (n = 118)		SD (n = 52)		p-value
		n	(%)	n	(%)	n	(%)	n	(%)		n	(%)	n	(%)	n	(%)	
Sex	Male	78	(45.1)	35	(44.9)	39	(50.0)	4	(23.5)	0.14	1	(33.3)	55	(46.6)	22	(42.3)	0.84 0.62*
	Female	95	(54.9)	43	(55.1)	39	(50.0)	13	(76.5)		2	(66.7)	63	(53.4)	30	(57.7)	
Age (years)	[2–5]	48	(27.8)	25	(32.0)	17	(21.8)	6	(35.3)	0.17	1	(33.3)	33	(28.0)	14	(26.9)	0.23 0.11*
	]5–10]	90	(52.0)	34	(43.6)	46	(59.0)	10	(58.8)		2	(66.7)	56	(47.4)	32	(61.5)	
	]10–15]	35	(20.2)	19	(24.4)	15	(19.2)	1	(5.9)		0	-	29	(24.6)	6	(11.6)	
Dengue immune status^§^	Primary	29	(16.8)	21	(26.9)	8	(10.3)	0	-	**7.4e-4**	1	(33.3)	27	(22.9)	1	(1.9)	**2.8e-5** 9.7e-5*
	Secondary	127	(73.4)	45	(57.7)	66	(84.6)	16	(94.1)		0	-	77	(65.2)	50	(96.2)	
Dengue infection	DENV-1	162	(93.6)	76	(97.4)	69	(88.5)	17	(100)	nt	3	(100)	111	(94.1)	48	(92.3)	nt
	DENV-2	2	(1.2)	2	(2.6)	0	-	0	-	nt	0	-	1	(0.8)	1	(1.9)	nt
	NS1 only	5	(2.9)	0	-	5	(6.4)	0	-	nt	0	-	2	(1.7)	3	(5.8)	nt
	Dengue IgM seroconversion	4	(2.3)	0	-	4	(5.1)	0	-	nt	0	-	4	(3.4)	0	-	nt
Admission day after onset of fever (±SD; median)		3.5 (±1.4; 4)		3.0 (±1.3; 3)		3.8 (±1.2; 4)		4.6 (±1.4; 5)		**5.3e-7**^$^	1.7 (±1.2; 1)		3.3 (±1.3; 3)		4.0 (±1.5; 4)		**1.2e-3**^$^
Mean number of days of hospitalization (±SD; median)		4.5 (±1.2; 4)		4.6 (±1.1; 4)		4.5 (±1.2; 4)		4.5 (±1.5; 4)		0.77^$^	4.0 (±1.0; 4)		4.6 (±1.2; 4)		4.4 (±1.1; 4)		0.61^$^

DF, dengue fever; DHF, dengue hemorrhagic fever; DSS, dengue shock syndrome; D-nonWS, dengue without warning signs; DWWS, dengue with warning signs; SD, severe dengue; DENV, dengue virus; DENV-1, dengue virus serotype 1; DENV-2, dengue virus serotype 2; CHIKV, Chikungunya virus; NT, not tested.

^§^ Dengue immune status was determined using WHO guidelines and as described in the Methods section [14].

p-values were calculated using Fisher exact tests except ^$^ one-way ANOVA. The latter was significant (p<0.01) for *Admission day after onset of fever*, so we then performed post-hoc Tukey tests on this variable; the following pairwise differences were significant: *WHO 1997 classification*: DF vs. DHF, p = 2.2e-4; DF vs. DSS, p = 6.3e-6; *adapted WHO 2009 classification*: D-nonWS vs. SD, p = 0.01; DWWS vs. SD, p = 0.01.

* indicates an additional Fisher exact test performed on only the DWWS and SD patients.

### Other arbovirus infections

In addition to the laboratory-confirmed dengue cases, 33 other arbovirus infections (OAI) were diagnosed as follows: acute Chikungunya infection (positive CHIKV RT-qPCR, n = 12; CHIKV-specific IgM seroconversion, n = 1), acute dengue and Chikungunya coinfection (n = 1), recent infection by Chikungunya (n = 1), recent infection by Chikungunya and dengue (n = 1), and recent infection by a flavivirus (n = 17) (S1 Table). Differences in clinical and laboratory parameters observed between DI and NDI patients were not always observed between DI and OAI patients (Table 1). This might be expected considering the combination of different recent arboviruses infections and uncertainty in the final diagnosis due to serological cross-reactivity. Consequently, we decided to compare only DI subjects with NDI (including non-dengue/non-arbovirus subjects) in the subsequent analyses.

### Comparison of WHO 1997 and 2009 criteria

In order to compare the WHO 1997 and 2009 classification criteria, we performed strict classifications of the of the 173 laboratory-confirmed dengue patients as outlined in the classification schemes in Methods and S1 Appendix. According to the WHO 1997 classification scheme, 78 were classified as DF, 78 as DHF, and 17 as DSS. Using the adapted WHO 2009 case definition, the 173 laboratory-confirmed dengue patients were classified as 3 D-nonWS (1.7%), 118 DWWS (68.2%), and 52 SD (30.1%) (Tables 2 and 3).

**Table 3:** Comparison of WHO 1997 and adapted WHO 2009 dengue classifications among hospitalized children with laboratory-confirmed dengue diagnoses (n = 173).

			Adapted WHO 2009 classification							
			D-nonWS		DWWS		SD		Total	
			n	(%)	n	(%)	n	(%)	n	(%)
**WHO 1997 classification**	DF	(45.1%^a^)	3	(3.8%)	68	(87.2%)	7	(9.0%)	**78**	**(100%)**
	DHF	(45.1%^a^)	0	(0%)	50	(64.1%)	28	(35.9%)	**78**	**(100%)**
	DSS	(9.8%^a^)	0	(0%)	0	(0%)	17	(100%)	**17**	**(100%)**
	**Total**		**3**	**(1.7%**^**a**^**)**	**118**	**(68.2%**^**a**^**)**	**52**	**(30.1%**^**a**^**)**	**173**	**(100%)**

DF, dengue fever; DHF, dengue hemorrhagic fever; DSS, dengue shock syndrome; DWWS, dengue with warning signs; SD, severe dengue.

^a^ Percentage of each dengue clinical presentation observed out of the total number of laboratory-confirmed dengue patients.

Comparing both classification schemes, only 3.8% (n = 3) of the DF met the D-nonWS criteria, while 87.2% (n = 68) matched with DWWS and 9.0% (n = 7) with SD (Table 3). Among the 78 DHF cases, 64.1% (n = 50) were classified as DWWS and the remaining cases as SD (35.9%, n = 28). All DSS cases were classified as SD (n = 17). Note that no cases with severe bleeding were included in this study.

Table 4 shows the discrepancies in the clinical manifestations of WHO 1997-classified DF patients (n = 78) compared to the revised WHO 2009 case definitions, including the main clinical signs observed with both classification schemes. Among the 61 (78.2%) DF patients presenting hemorrhagic tendencies, 16 had these alone and were classified as DWWS (n = 15) or D-nonWS (n = 1), while 32 had thrombocytopenia as well, and were classified DWWS under the WHO 2009 criteria. Furthermore, 11 DF had hemorrhagic tendencies and plasma leakage signs and were classified as DWWS (n = 10) or SD (n = 1). One DF patient had plasma leakage and signs of cardiovascular shock, thus corresponding to the SD category, and one had an isolated sign of shock classified as DWWS.

**Table 4:** Description of hemorrhagic and shock clinical manifestations presented by laboratory-confirmed dengue patients classified as DF (n = 78) using the WHO 1997 case definition, with comparison to the adapted WHO 2009 dengue classification.

		n (%)	Thrombocytopenia ≤ 100x10^9^/L	Fluid accumulation signs	Signs of shock	Comparison to the adapted WHO 2009 classification
**DF** **n = 78**	With hemorrhagic tendency n = 61 (78.2%)	16* (26.2%)				**D-nonWS** (n = 1) **DWWS** (n = 15)
		32 (52.5%)	✔^¶^			**DWWS** (n = 32)
		11 (18.1%)		✔^$^		**DWWS**: including mild plasma leakage (n = 10) **SD**: plasma leakage with respiratory distress (n = 1)
		1 (1.6%)		✔	✔	**SD**: all signs of shock with plasma leakage and respiratory distress were observed at admission
		1 (1.6%)			✔	**DWWS**: isolated sign of shock (narrow pulse pressure at defervescence; hepatomegaly detected by ultrasonographic examination)
	Without hemorrhagic tendency n = 17 (21.8%)	4 (23.5%)	✔^¥^			**D-nonWS** (n = 2) **DWWS** (n = 2)
		5 (29.4%)	✔^¥^	✔^§^		**DWWS**: including mild plasma leakage (n = 4) **SD**: plasma leakage with respiratory distress (n = 1)
		2 (11.8%)		✔^#^		**DWWS**: including mild plasma leakage (n = 2)
		5 (29.4%)	✔^¥^	✔^##^	✔	**DWWS**: isolated sign of shock (narrow pulse pressure) with mild plasma leakage at admission (n = 1) **SD**: plasma leakage and respiratory distress (n = 3); mild plasma leakage associated with signs of shock (n = 1)
		1** (5.9%)				**DWWS**: abdominal tenderness at defervescence

DF, dengue fever; D-nonWS, dengue without warning signs; DWWS, dengue with warning signs; SD, severe dengue.

* Patient classified as DF with an isolated hemorrhagic tendency.

** Patients classified as DF without clinical signs of either DHF or DSS.

^¶^ Thrombocytopenia (x10^9^/L): mean: 70; 95% CI: 64.4–74.6 (n = 32).

^¥^ Thrombocytopenia (x10^9^/L): mean: 51; 95% CI: 40.9–61.9 (n = 14).

^$^ Ultrasonographic examination of the abdomen with positive findings for all DF cases (n = 11); additionally, two patients presented high hematocrit levels (more than 20% above average for their age) at Visit 1 followed by a drop in hematocrit levels between two visits greater than 20%.

^§^ Ultrasonographic examination of the abdomen with positive findings for all DF cases (n = 5); additionally, two patients had increased hematocrit greater than 20% above average for age at Visit 1 followed by a drop of hematocrit levels between two visits greater than 20%.

^#^ Ultrasonographic examination of the abdomen with positive findings for the two DF cases.

^##^ Ultrasonographic examination of the abdomen with positive findings for all DF cases (n = 5); additionally, four patients had an increased hematocrit levels greater than 20% above average for age at Visit 1 associated with a drop of hematocrit level between 2 visits greater than 20%.

Of the 17 (21.8%) DF patients without hemorrhagic tendencies, 4 had isolated thrombocytopenia (classified as 2 D-nonWS and 2 DWWS), 5 had thrombocytopenia and plasma leakage (4 DWWS and 1 SD), 2 had only plasma leakage and were classed as DWWS, and 5 had thrombocytopenia with plasma leakage and signs of shock, meaning they were classified as DWWS with an isolated sign of shock (n = 1) or SD (n = 4). Finally, 1 DF patient did not exhibit any signs of severity, but had abdominal tenderness at defervescence and was classified as DWWS.

In the set of DHF patients (n = 78) as classified by the WHO 1997 classification scheme, 28 (35.9%) presented clinical signs of SD, including pleural effusion–quantified by ultrasound as moderate or abundant (n = 26)–and/or elevated AST, over 1000 IU/L (n = 2). The 50 (64.1%) remaining DHF cases were identified as DWWS using the WHO 2009 case definition. Clinical warning signs were observed in at least 44 children at Visit 1 and in 4 additional patients at Visit 2. Using ultrasound, 43 patients had fluid accumulation: ascites fluid alone (n = 19), ascites associated with pleural effusion (n = 17), or isolated pleural effusion (n = 7), were observed during at least one visit. Additionally, an increase in hematocrit above the normal range, concurrent with thrombocytopenia, was observed in 8 patients at different time points: Visit 1 (n = 4) and Visit 2 (n = 4). Finally, a drop in platelet count of 50% or more between two consecutive visits occurred in 17 patients.

A pre-discharge/discharge visit for patients who recovered entirely, or a follow-up visit for patients in the critical phase, corresponded to Visit 3. Table 5 presents the distribution of dengue patients in the critical phase at Visit 3 with follow-up, according to their WHO 1997 and adapted WHO 2009 dengue classifications. Among all DI (n = 173), 39 (22.5%) had a follow-up visit; this corresponded to 87.1% of the DHF/DSS patients, and 53.8% of the SD ones. Of note, Visit 3 follow-ups for patients in the critical phase usually occurred earlier than for discharged ones.

**Table 5:** Distribution of dengue patients in the critical phase at Visit 3 with follow-up, with respect to their WHO 1997 and adapted WHO 2009 dengue classifications.

	Admission	Visit 3 with clinical/biological monitoring or critical phase		Comparison with adapted WHO 2009 classification
	n	n	(%)	
DF	78	5	(6.4%)	1 DWWS; 4 SD
DHF	78	29	(37.2%)	17 DWWS; 12 SD
DSS	17	5	(29.4%)	5 SD

DF, dengue fever; DHF, dengue hemorrhagic fever; DSS, dengue shock syndrome; DWWS, dengue with warning signs; SD, severe dengue.

Finally, among all SD (n = 52) classified with adapted WHO 2009 by using ultrasound, all but 2 patients (96%) presented plasma leakage characterized by at least moderate or abundant pleural effusion at one or two visits, associated or not with plasma leakage based on hematocrit fluctuation, signs of shock or severe organ involvement. Description of biological (i.e. plasma leakage based on hematocrit fluctuation) and clinical manifestations among SD is presented in Table 6.

**Table 6:** Description of biological and clinical manifestations presented by laboratory-confirmed dengue patients classified as SD (n = 52) adapted from the WHO 2009 case definition.

		n (%)	Plasma leakage based on ultrasound	Signs of shock	Severe organ involvement
**SD** **n = 52**	With plasma leakage based on hematocrit variation n = 21 (40.4%)	15 (71.4%)	✔*		
		6 (28.6%)	✔^¶^	✔	
	Without plasma leakage based on hematocrit variation n = 31 (59.6%)	16 (51.5%)	✔^#^		
		9 (29.0%)	✔^#^	✔	
		2 (6.5%)		✔	
		2 (6.5%)	✔^#^	✔	✔^§^
		2 (6.5%)	✔^#^		✔^§^

* Presence of ascites with moderate pleural effusion at one or two visits.

^¶^ Presence of mild or moderate pleural effusion at one or two visits.

^#^ Presence of ascites with moderate or abundant pleural effusion at one or two visits.

^§^ Aspartate Aminotransferase > 1,000 IU/L.

### Biological parameters differing between DF, DHF, and DSS, and between DWWS and SD

#### Dengue viral load and NS1 antigen

**Fig 1:**
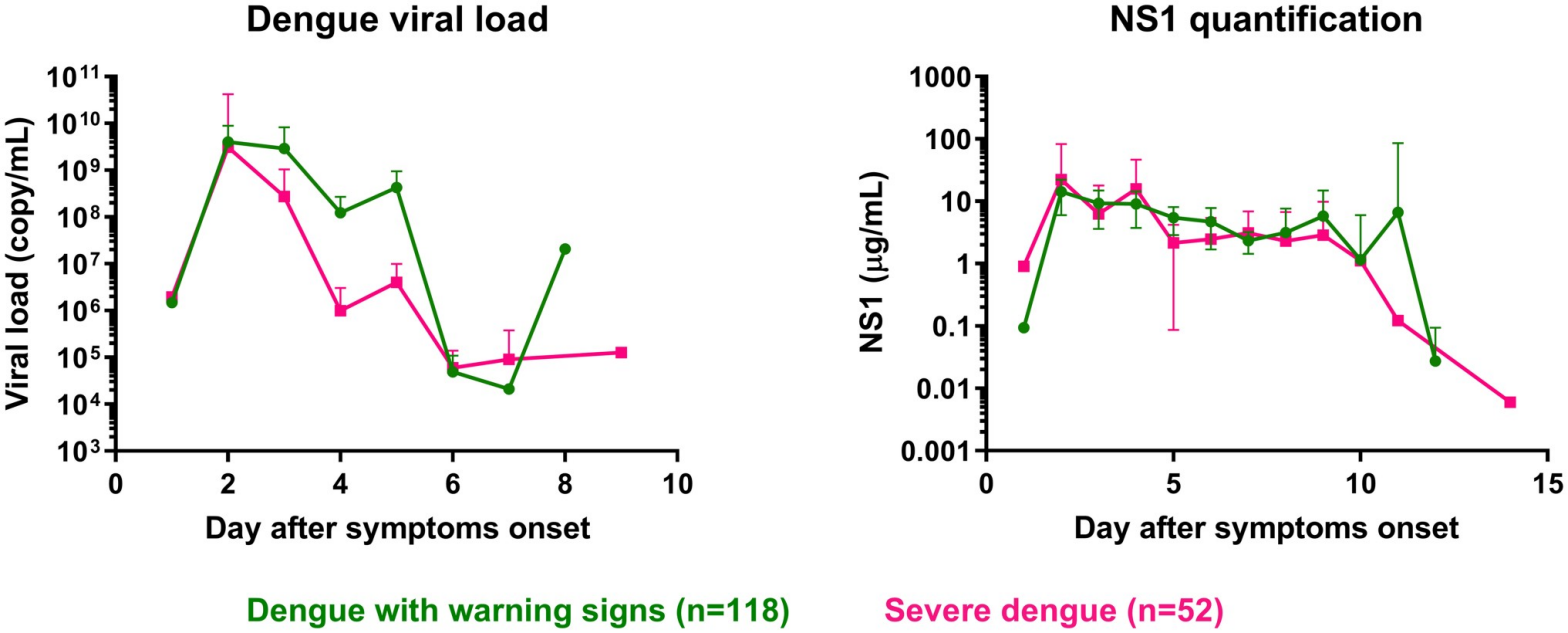
Dengue viral load at admission and NS1 antigen levels during hospitalization in dengue-infected patients using the adapted WHO 2009 classification scheme. Dengue with warning signs (DWWS; green circles), severe dengue (SD; pink squares). Error bars correspond to the 95% confidence interval around the mean.

We evaluated differences in viral load and NS1 concentration at admission between different dengue severity groups (Fig 1 and S1 Fig). Here, the mean viral load pooled over days 3–5 in SD patients (4.5 Log_10_ copy/mL, 95% CI: 3.8–5.2) was lower than in DWWS patients. Additionally, the average NS1 concentration pooled over days 1–14 in SD patients was lower than in DWWS patients. Considering the WHO 1997 classification, there was no difference in viral load at any day after onset of symptoms. The NS1 concentration pooled over the whole range of admission days (day 1–14) was significantly lower in DHF/DSS patients (5.6 μg/mL, 95% CI: 3.3–7.8) than in DF patients (5.8 μg/mL, 95% CI: 4.3–7.3; p = 1.1e-3; S1 Fig).

#### Hematological parameters

**Fig 2:**
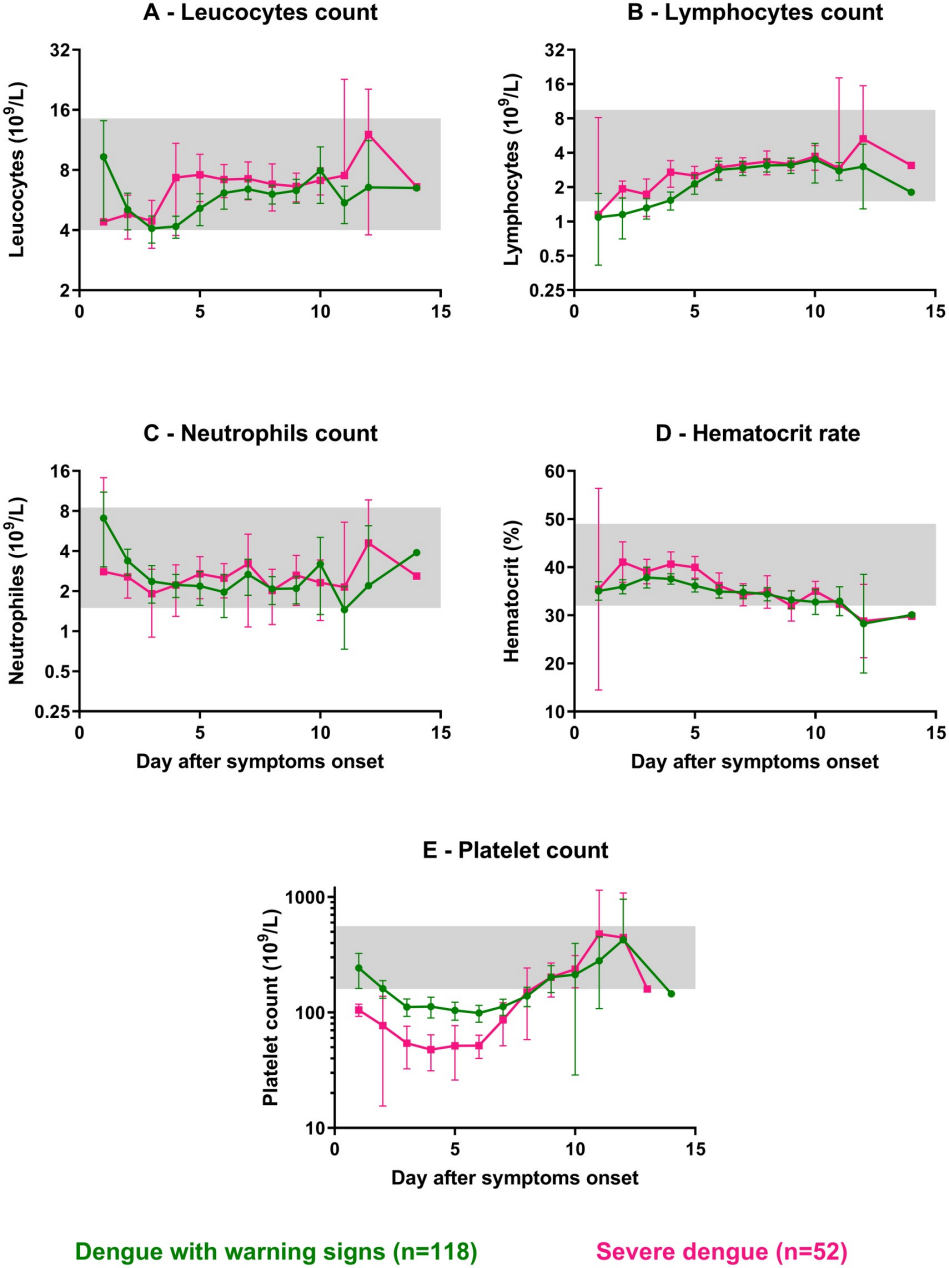
Hematological parameters observed in hospitalized dengue-infected patients for the adapted WHO 2009 classification scheme. Values from admission, deferverscence, and discharge visits for: dengue with warning signs (DWWS; green circles), severe dengue (SD; pink squares). (**A**) Leucocytes count (10^9^/L); (**B**) Lymphocytes count (10^9^/L); (**C**) Neutrophils count (10^9^/L); (**D**) Hematocrit rate (%); (**E**) Platelet count (10^9^/L). Each circle or square represents the mean of each variable's observed values per day. Error bars correspond to 95% confidence intervals around the mean. Grey areas represent the normal range for each parameter. Normal ranges used for hematological parameters were obtained for children aged from 2 to 15 years old from: http://www.hematocell.fr/index.php/les-cellules-du-sang/15-les-cellules-du-sang-et-de-la-moelle-osseuse/valeurs-normales-de-lhemogramme-selon-lage/129-hemogramme-selon-lage.

A decrease in platelet count and increased hematocrit concentrations are included in both the WHO 1997 and adapted WHO 2009 classification criteria. Here, we investigated to what extend they separate differently classified patients and which dynamics they follow over time in the various patient populations. The mean platelet count pooled over days 3–6 in SD patients (51x10^9^/L, 95% CI: 41–61) was significantly below that of DWWS (106x10^9^/L, 95% CI: 96–116; p = 5.4e-14, Mann-Whitney test; Fig 2). Moreover, both means were below the platelet lower normal range (<160x10^9^/L). Additionally, the mean platelet count pooled over days 3–6 in DHF/DSS patients (62x10^9^/L, 95% CI: 54–71) was significantly below that of DF (127x10^9^/L, 95% CI: 115–139; p<1e-15, Mann-Whitney test–S2 Fig).

#### Biochemical parameters

**Fig 3:**
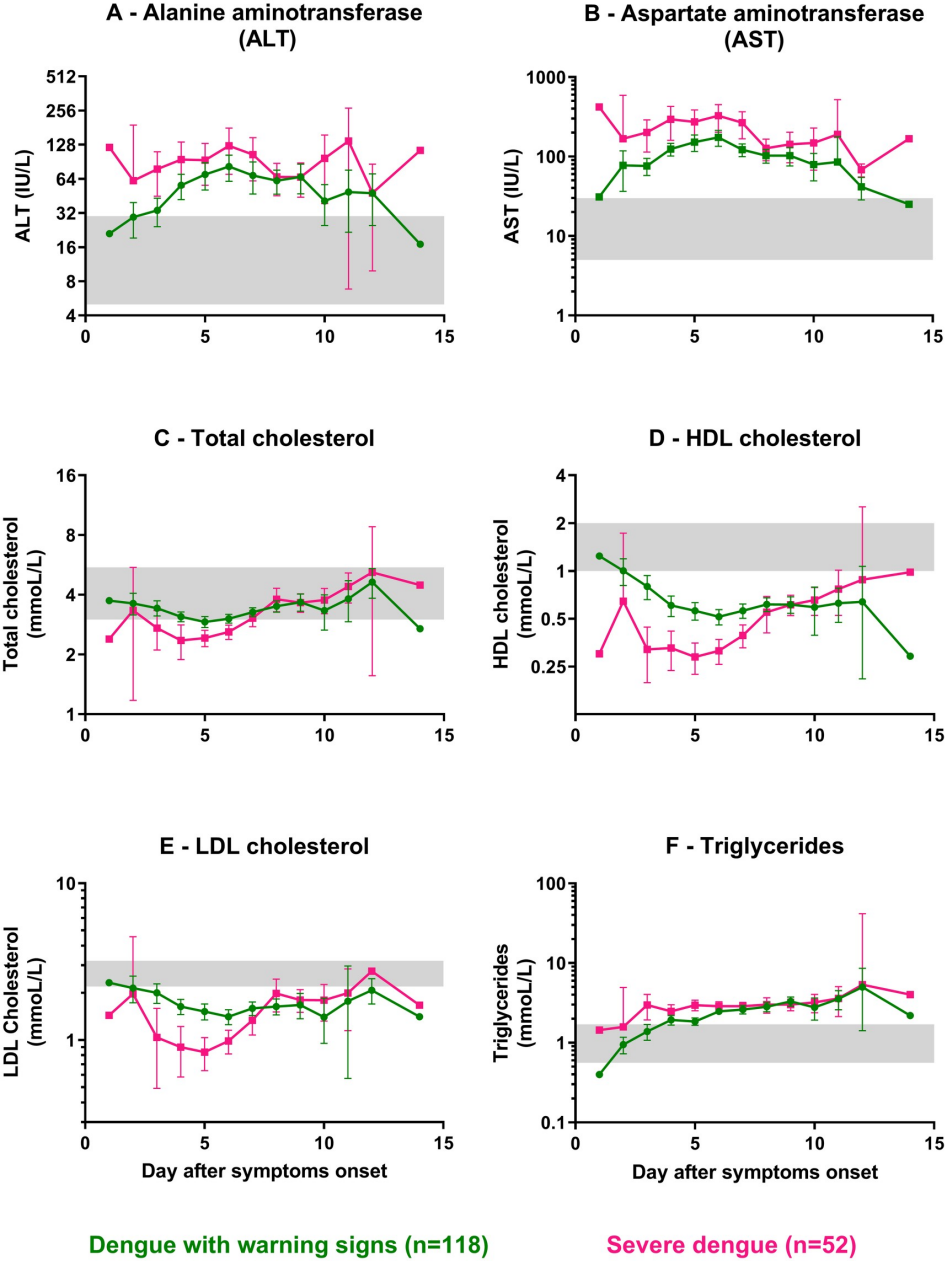
Biochemical parameters observed in hospitalized dengue-infected patients using the adapted WHO 2009 classification scheme. Values at admission, deferverscence and discharge for: dengue with warning signs (DWWS; green circles), severe dengue (SD; pink squares). *Liver function*: (**A**) Alanine aminotransferase (ALT; IU/L); (**B**) Aspartate aminotransferase (AST; IU/L); *Lipid profile*: (**C**) Total cholesterol (mmol/L); (**D**) HDL cholesterol (mmol/L); (**E**) Calculated LDL cholesterol (mmol/L); (**F**) Triglycerides (mmol/L). Each circle or square represents the mean of each variable's observed values per day. Error bars correspond to 95% confidence intervals around the mean. Grey areas represent the normal range for each parameter. Normal ranges used for biochemical parameters were obtained from: https://emedicine.medscape.com/article/2172316-overview.

We evaluated differences in the most impacted biochemical parameters along the course of dengue illness between different dengue severity groups. For liver function, the mean ALT in SD patients was significantly higher than for DWWS patients over days 1–3 pooled together. Furthermore, combining all admission days (1–14), the mean ALT was twice the upper normal range (63 IU/L, 95% CI: 56–69) in DWWS patients and was significantly higher than this in SD patients, with a mean three times the upper normal range (96 IU/L, 95% CI: 81–112; p = 7.3e-10, Mann-Whitney test). Similarly, the mean AST in SD patients was significantly higher than for DWWS patients over days 1–4, and the mean AST in DWWS and SD patients over combined days 1–14 were four (124 IU/L, 95% CI: 113–135) and eight (239 IU/L, 95% CI: 201–278; p<1e-15, Mann-Whitney test) times the upper normal range, respectively (Fig 3). As for the WHO 1997 classification, combining days 1–14, the mean ALT was twice the upper normal range (57 IU/L, 95% CI: 49–65) in DF patients and was significantly higher in DHF/DSS patients combined, with a mean around three times the upper normal range (85 IU/L, 95% CI: 75–95; p = 7e-10, Mann-Whitney test). The mean AST in DF and DHF/DSS patients over combined days 1–14 were three (114 IU/L, 95% CI: 100–127) and more than six times the upper normal range (194 IU/L, 95% CI: 170–217), respectively (S3 Fig), and this difference was significant (p = 5e-15, Mann-Whitney test).

#### Lipid profiles

Recent studies have reported that host lipids like cholesterol are modulated by flaviviruses during infection in mammals, and that changes in lipid profiles are associated with severe forms of dengue in humans [38,39]. A comparison of lipid parameters over the hospitalization period provided significant results: the mean total cholesterol in SD patients (2.5 mmol/L) was below the lower normal range (i.e., <3 mmol/L) early in the disease course (day 3–6) and significantly below the mean total cholesterol in DWWS patients (3.1 mmol/L, 95% CI: 3.0–3.2, p = 7.8e-12, t-test) over the same period (Fig 3). A similar pattern was observed for HDL cholesterol, with even more striking differences. Mean HDL cholesterol values were below the lower normal range (<1 mmol/L) for both categories across all days after symptoms onset. In addition, HDL cholesterol in SD patients (0.33 mmol/L, 95% CI: 0.30–0.36) was significantly below that of DWWS patients (0.58 mmol/L, 95% CI: 0.55–0.62, p<1e-15 Mann-Whitney test) for the pooled data from days 3–7 (Fig 3). In parallel, the mean LDL cholesterol in SD patients (0.92 mmol/L, 95% CI: 0.81–1.0) was significantly below that in the DWWS patients (1.6 mmol/L, 95% CI: 1.5–1.7, p = 4.4e-14, Mann-Whitney test) in pooled data over days 3–6 (Fig 3). Triglycerides behaved differently, with means increasing above the upper normal range (i.e., >1.7 mmol/L) during hospitalization in DWWS and SD patient groups after 4 days of symptoms (Fig 3). The mean triglycerides levels pooled over days 1–5 in DWWS (1.7 mmol/L, 95% CI: 1.5–1.8) and SD (2.7 mmol/L, 95% CI: 2.4–3.1) patients were significantly different (p = 1.7e-9, Mann-Whitney test).

The current study was conducted in the field in the Cambodian context in a real life setting, where the day of hospitalization with respect to onset of disease was quite variable (Table 1). This situation is not uncommon in many countries where dengue is endemic. Hence, we were interested in evaluating various biochemical parameters at hospital admission rather than day of illness in order to inform clinicians working in these settings. The comparison of the evolution of lipid profile parameters from visit to visit in the DI and NDI groups is shown in S4 and S5 Figs. Working under the adapted WHO 2009 classification scheme, total cholesterol, HDL, and LDL cholesterol levels in the DI and NDI groups all evolved in a similar way over time, first with low values compared to the normal, then gradually moving back up towards the normal. As for triglycerides, their levels were elevated at admission with compared to the normal, tending to rise higher at defervescence before gradually dropping back towards the normal. Similar trends were observed between the DF, DHF, and DSS groups as between DWWS and SD (S6 and S7 Figs).

## Discussion

Dengue disease presents as a continuous range rather than distinct clinical entities and much of the time has an unpredictable clinical outcome. In particular, some patients will not develop severe dengue and thus do not require hospitalization. WHO criteria were set up to help clinicians classify patients in order to reduce hospital work loads and provide appropriate patient management. Previous studies comparing the two classification schemes observed that the WHO 1997 one's DF/DHF/DSS division of patients is poorly related to disease severity, is difficult to apply prospectively in current practice, is not very helpful for triage in outbreaks, and leads to different reporting worldwide due to difficulties clinicians find in using it [26]. As for the WHO 2009 classification, defining categories with warning then severity signs (DWWS/SD) has the advantage of being able to define the severity of dengue cases in a timely manner, facilitating dengue case management and surveillance, thus helping to reduce dengue mortality. However, clinical criteria are less strictly defined in it, leaving room for arbitrary interpretation by clinicians [22,23,25].

In this study we compared how well the two classification schemes–WHO 1997 classification versus an adapted WHO 2009 classification for SD case definition with systematic use of ultrasound–correctly classify dengue severity, as well as their usefulness in dengue patient management, in a pediatric cohort of hospitalized severe dengue patients in Cambodia. Recorded values of well-characterized symptoms as used in both classifications: biochemical values, ultrasonography data associated with a reliable diagnosis of dengue infection based on genome and antigen detection or antibody seroconversion detection, were consistently and carefully reported on regular basis throughout the hospitalization period so that the dengue severity classifications were as accurate as possible.

Although the well-characterized pediatric population with clinical and biological parameters confers strength to our data, our study may suffer from limitations. Firstly, only two DENV serotypes were detected (DENV-1 and DENV-2) with DENV-1 largely predominant. Secondly, we defined DI group with including children with laboratory evidence of dengue infection and OAI group of infected patients with inconclusive confirmed arbovirus infection due to serological cross-reactivity between flaviviruses (beside one DENV/CHIV coinfection). We decided to not consider patient from OAI from our analysis to reduce the risk of bias, as including the DENV/CHIKV co-infection and others probable recent flavivirus infections might have an impact on results.

Similar to what has been previously demonstrated in other countries, in this cohort of Cambodian children we observed that the WHO 1997 classification acts longitudinally, using strict prerequisite clinical and/or biological signs to move from one step to the next [16,24,26]. Thus, a patient with all DSS clinical signs cannot be classified as DSS if they do not first meet all clinical and/or laboratory criteria for DF and DHF. This can lead to underestimating the number of severe dengue cases and inadequate clinical management. In our study, 87.2% and 9% of DF cases were actually classified respectively as DWWS and SD, and 35.9% of DHF were considered as SD using the adapted WHO 2009 classification. Interestingly, all 7 patients classified as DF according to the WHO 1997 case definition and classified as SD according to adapted WHO 2009, showed at least some sign of plasma leakage, highlighting the discrepancy observed between these two classifications. Additionally, SD more precisely defines the group of patients requiring intensive clinical follow-up, since SD includes all of the DSS plus 36% of the DHF, and even 9% of the DF, in our study. However, we adapted the WHO 2009 dengue classification for defining SD by using ultrasound to anticipate an aggravation of the patient with occurrence of respiratory distress. This strategy might explain the high proportion of SD case reported during this study. While using the strict 2009 criteria cases with moderate or large pleural effusion but without clinical respiratory distress would be classified as DWWS, so the discrepancies observed between the two systems would be less marked. Though the 1997 classification seems poorly-adapted to early and appropriate patient clinical management, its separation into different dengue clinical syndromes probably makes it more suitable for the study of dengue pathogenesis.

The WHO 2009 case definition, being transversal with a broad definition of the different forms of dengue fever in the DWWS category, is more flexible in terms of clinical management of hospitalized dengue-confirmed cases [40]. Clinicians may independently consider clinical and biological criteria related to hemorrhagic tendencies and plasma leakage as warning signs, and thus adapt appropriate patient management at given times during hospitalization. Moreover, besides dengue shock syndrome, the WHO 2009 classification proposes definitions for non-DHF types of severe dengue fever with severe organ involvement [11], leading to an emphasis on triage and clinical management, rather than on a pathophysiologically defined syndrome [26].

Our results demonstrate that the systematic use of ultrasonographic examination at the time of patient admission is important to evaluate plasma leakage as early as possible. Ultrasonograms are more sensitive, more accurate, and more practical for detecting plasma leakage than the 20% increase in hematocrit and/or lateral decubitus chest X-ray recommended by WHO 1997, and provide objective evidence. Moreover, serial ultrasonographic examinations can be conveniently repeated regularly during hospitalization. This has previously shown its effectiveness [41] in contrast to studies where ultrasound examinations were performed only once [42,43]. Moreover, most severe dengue cases are hospitalized close to the critical phase, i.e., 4 to 5 days after onset of symptoms, and hence need accurate and immediate clinical management. Importantly, our findings highlight that the presence of at least one plasma leakage sign at admission (ascites or pleural effusion, both detected by ultrasound, or facial oedema observed during clinical examination) could significantly help differentiate dengue case from other OAI and NDI. This suggests that detection of plasma leakage at admission using ultrasound, in association with other clinical and biological parameters, should be further explored as a potential diagnostic tool and prognosis marker of dengue severity.

Laboratory changes were well-characterized in our study in dengue patients at different stages of the disease. These included classical hematological and biochemical parameters (liver function and inflammation), dengue viral load and NS1 antigen concentration. As previously reported, transaminase levels were significantly higher and platelet count lower in SD patients compared to DWWS patients. Levels of secreted NS1 pooled over all days after onset of symptoms were lower in the more severe cases in both classification schemes (SD vs DWWS and DHF/DSS vs DF, respectively). Interestingly, lipid profile changes evaluated in our study were of great relevance, with differences between DWWS and SD seen in particular at hospital admission. Changes in lipid levels were observed mainly in SD patients, characterized by a decrease in total cholesterol, HDL and LDL cholesterol at admission. Although there is a trend towards a return to normal total cholesterol at hospital discharge, HDL and to a lesser extent LDL cholesterol levels remain below the normal range for both DWWS and SD.

Total cholesterol consists mostly of LDL cholesterol and HDL cholesterol and, to a lesser extent, very low-density lipoprotein (VLDL) cholesterol and triglycerides. A relationship between circulating lipids and dengue infection has been previously observed in studies, with lower levels of total cholesterol seen in severe dengue cases than in less severe ones or in healthy people [44,45]. Moreover, two previous studies described a decrease in total and LDL cholesterol levels during dengue disease, with the size of the decrease being correlated with the severity of the disease [38,46]. These results are in partial agreement with our observations, as the decrease in HDL cholesterol observed in our study seems to be as much or even more significant as that of LDL.

However, the relationships between severe dengue and total, HDL, and LDL cholesterol, respectively, remain unclear. It has been reported that during dengue infection, the modulation of levels of host cholesterol facilitates viral entry, formation of replicative complexes, viral assembly, and control of the interferon type I response [39]. Moreover, the dengue sNS1 protein is a nonstructural protein that is secreted in blood during patient viremia and forms an atypical high density lipoprotein particle, suggesting that dengue NS1 might mimic or hijack lipid metabolic pathways and contribute to endothelium dysfunction [47]. These observations together suggest that interactions between NS1 protein and lipid metabolism might be involved in dengue pathogenesis.

In conclusion, our results bolster previous reports regarding discrepancies in the classification of severe dengue case between the WHO 1997 and 2009 guidelines due to the stepwise organization of clinical and/or biological signs used in the 1997 classification. Using an adapted WHO 2009 classification, SD more precisely defined the group of patients requiring clinical follow-up during hospitalization Thus, the most appropriate therapy can be quickly initiated. Finally, the challenge remains to predict the outcome of dengue-infected. The systematic use of ultrasonographic examination at admission is crucial in order to evaluate plasma leakage. In addition, lipid markers are potential predictive markers of dengue severity at hospital.

## Supporting information

S1 Checklist STROBE statement. (PDF)

S1 Appendix Description of dengue classifications used following WHO 1997 and WHO 2009 guidelines. (PDF)

S1 Table Interpretation criteria of virological and serological analyzes to define acute dengue infection (DI), other arbovirus infection (OAI) and non-dengue infection (NDI) groups. (PDF)

S1 Fig Dengue viral load and NS1 antigen quantification in dengue-infected patients during hospitalization using the WHO 1997 classification scheme. Dengue fever (DF; blue circles), dengue hemorrhagic fever (DHF; red squares), dengue shock syndrome (DSS; orange triangles). Error bars correspond to the 95% confidence interval around the mean.
(TIF)

S2 Fig Hematological parameters observed in hospitalized dengue-infected patients using the WHO 1997 classification scheme. Hematological parameter values observed in dengue-infected patients during hospitalization classified as: dengue fever (DF; blue circles), dengue hemorrhagic fever (DHF; red squares) or dengue shock syndrome (DSS; orange triangles). (**A**) Leucocyte count (10^9^/L); (**B**) Lymphocyte count (10^9^/L); (**C**) Neutrophils count (10^9^/L); (**D**) Hematocrit rate (%); (**E**) Platelet count (10^9^/L). Each circle/square/triangle represents the mean of each variable's observed values per day. Error bars correspond to 95% confidence intervals around the mean. Grey areas represent the normal range for each parameter. Normal ranges used for hematological parameters were obtained for children aged from 2 to 15 years old from: http://www.hematocell.fr/index.php/les-cellules-du-sang/15-les-cellules-du-sang-et-de-la-moelle-osseuse/valeurs-normales-de-lhemogramme-selon-lage/129-hemogramme-selon-lage.
(TIF)

S3 Fig Biochemical parameters observed in hospitalized dengue-infected patients using the WHO 1997 classification scheme. Biochemical parameter values observed in dengue-infected patients during hospitalization classified as: dengue fever (DF; blue circles), dengue hemorrhagic fever (DHF; red squares) or dengue shock syndrome (DSS; orange triangles). *Liver function*: (**A**) Alanine aminotransferase (ALT; IU/L); (**B**) Aspartate aminotransferase (AST; IU/L); *Lipid profile*: (**C**) Total cholesterol (mmol/L); (**D**) HDL cholesterol (mmol/L); (**E**) Calculated LDL cholesterol (mmol/L); (**F**) Triglycerides (mmol/L). Each circle/square/triangle represents the mean of each variable's observed values per day. Error bars correspond to a 95% confidence interval around the mean. Grey areas represent the normal range for each parameter. Normal ranges used for biochemical parameters were obtained from: https://emedicine.medscape.com/article/2172316-overview.
(TIF)

S4 Fig Triglycerides and total cholesterol levels in dengue-infected patients using the adapted WHO 2009 classification scheme, and non-dengue infected patients. Comparison of triglyceride (**A**) and total cholesterol (**B**) levels in dengue-infected patients using the adapted WHO 2009 classification scheme and non-dengue infected patients (excluding other arbovirus infections) at admission, defervescence and discharge (or follow-up) visits. Dengue with warning signs: green circles; Severe dengue: pink circles; Non-dengue infection: black circles. Sampling day ranges are presented on the *x*-axis for each group. Mean triglycerides and total cholesterol levels shown, with error bars indicating the standard deviation. Asterisks indicate statistically significant differences in mean triglycerides and total cholesterol levels between different patient categories (** p<0.01, **** p<0.0001; one-way ANOVA followed by Tukey’s post hoc test). Grey areas represent the normal range for triglyceride (0.56–1.70 mmol/L) and total cholesterol (3–5.5 mmol/L) levels.
(TIF)

S5 Fig HDL cholesterol and LDL cholesterol levels in dengue-infected patients using the adapted WHO 2009 classification scheme, and non-dengue infected patients. Comparison of HDL cholesterol (**A**) and LDL cholesterol (**B**) levels in dengue-infected patients using the adapted WHO 2009 classification scheme and non-dengue infected patients (excluding other arbovirus infections) at admission, defervescence and discharge (or follow-up) visits. Dengue with warning signs: green circles; Severe dengue: pink circles; Non-dengue infection: black circles. Sampling day ranges are presented on the *x*-axis for each group. Mean HDL and LDL cholesterol levels shown, with error bars indicating the standard deviation. Asterisks indicate statistically significant differences in mean HDL and LDL cholesterol levels between different patient categories (** p<0.01, **** p<0.0001; one-way ANOVA followed by Tukey’s post hoc test). Grey areas represent the normal range for HDL cholesterol (1–2 mmol/L) and LDL cholesterol (2.2–3.2 mmol/L) levels.
(TIF)

S6 Fig Triglycerides and total cholesterol levels in dengue-infected patients using the WHO 1997 classification scheme, and non-dengue infected patients. Comparison of triglycerides (**A**) and total cholesterol (**B**) levels in dengue-infected patients using the WHO 1997 classification scheme and non-dengue infected patients (excluding other arbovirus infections) at admission, defervescence and discharge (or follow-up) visits. Dengue fever: blue circles; Dengue hemorrhagic fever: red circles; Dengue shock syndrome: orange circles; Non-dengue infections: black circles. Sampling day ranges are presented on the *x*-axis for each group. Mean triglycerides and total cholesterol levels shown, with error bars indicating the standard deviation. Asterisks indicate statistically significant differences in mean triglycerides and total cholesterol levels between different patient categories (** p<0.01, *** p<0.001, **** p<0.0001; one-way ANOVA followed by Tukey’s post hoc test). Grey areas represent the normal range for triglyceride (0.56–1.70 mmol/L) and total cholesterol (3–5.5 mmol/L) levels.
(TIF)

S7 Fig HDL cholesterol and LDL cholesterol levels in dengue-infected patients using the WHO 1997 classification scheme, and non-dengue infected patients. Comparison of HDL cholesterol (**A**) and HDL cholesterol (**B**) levels in dengue-infected patients using the WHO 1997 classification scheme and non-dengue infected patients (excluding other arbovirus infections) at admission, defervescence and discharge (or follow-up) visits. Dengue fever: blue circles; Dengue hemorrhagic fever: red circles; Dengue shock syndrome: orange circles; Non-dengue infections: black circles. Sampling day ranges are presented on the *x*-axis for each group. Mean HDL and LDL cholesterol level shown, with error bars indicating the standard deviation. Asterisks indicate statistically significant differences in mean HDL and LDL cholesterol levels between different patient categories (** p<0.01, *** p<0.001, **** p<0.0001; one-way ANOVA followed by Tukey’s post hoc test). Grey areas represent the normal range for HDL cholesterol (1–2 mmol/L) and LDL cholesterol (2.2–3.2 mmol/L) levels.
(TIF)
