# Supplementary material for: Comparison of dengue case classification schemes and evaluation of biological changes in different dengue clinical patterns in a longitudinal follow-up of hospitalized children in Cambodia
Source: PLoS Negl Trop Dis. 2020 Sep 14;14(9):e0008603. doi: 10.1371/journal.pntd.0008603 (PMC7515206; doi:10.1371/journal.pntd.0008603)
Supplement: S1 Checklist — (PDF) [file pntd.0008603.s001.pdf]

S1 Checklist. STROBE Statement — Checklist for reports of observational studies.

	Item No	Recommendation	Manuscript
**Title and abstract**	1	(*a*) Indicate the study's design with a commonly used term in the title or the abstract	Abstract, paragraph 2
		(*b*) Provide in the abstract an informative and balanced summary of what was done and what was found	Abstract, paragraph 2 and 3
**Introduction**			
Background/ rationale	2	Explain the scientific background and rationale for the investigation being reported	Introduction, paragraph 1 and 2
Objectives	3	State specific objectives, including any pre-specified hypotheses	Introduction, paragraph 3
**Methods**			
Study design	4	Present key elements of study design early in the paper	Methods: Study participants and Clinical methods sections
Setting	5	Describe the setting, locations, and relevant dates, including periods of recruitment, exposure, follow-up, and data collection	Methods: Study participants and Clinical methods sections
Participants	6	(*a*) Give the eligibility criteria, and the sources and methods of selection of participants	Methods: Study participants and Clinical methods sections
Variables	7	Clearly define all outcomes, exposures, predictors, potential confounders, and effect modifiers. Give diagnostic criteria, if applicable	Methods: Blood collection and laboratory analyses, Classification of arbovirus infections and non-dengue infections and WHO 1997 and 200 classification sections
Data sources/ measurement	8*	For each variable of interest, give sources of data and details of methods of assessment (measurement). Describe comparability of assessment methods if there is more than one group	N/A
Bias	9	Describe any efforts to address potential sources of bias	N/A
Study size	10	Explain how the study size was arrived at	N/A
Quantitative variables	11	Explain how quantitative variables were handled in the analyses. If applicable, describe which groupings were chosen and why	N/A
Statistical methods	12	(*a*) Describe all statistical methods, including those used to control for confounding	Methods: Statistical analysis section
		(*b*) Describe any methods used to examine subgroups and interactions	Methods: Statistical analysis section
		(*c*) Explain how missing data were addressed	N/A
		(*d*) If applicable, describe analytical methods taking account of sampling strategy	N/A

		(*e*) Describe any sensitivity analyses	N/A
**Results**			
Participants	13*	(a) Report numbers of individuals at each stage of study—eg numbers potentially eligible, examined for eligibility, confirmed eligible, included in the study, completing follow-up, and analysed	Results: Characteristics of included patients section
		(b) Give reasons for non-participation at each stage	N/A
		(c) Consider use of a flow diagram	N/A
Descriptive data	14*	(a) Give characteristics of study participants (eg demographic, clinical, social) and information on exposures and potential confounders	Results: Characteristics of included patients, Demographic parameters, Clinical parameters and Biological parameters sections; Table 1
		(b) Indicate number of participants with missing data for each variable of interest	N/A
Outcome data	15*	Report numbers of outcome events or summary measures	Results: Laboratory-confirmed dengue infections and Other arbovirus infections sections; Table 2
Main results	16	(*a*) Give unadjusted estimates and, if applicable, confounder-adjusted estimates and their precision (eg, 95% confidence interval). Make clear which confounders were adjusted for and why they were included	N/A
		(*b*) Report category boundaries when continuous variables were categorized	N/A
		(*c*) If relevant, consider translating estimates of relative risk into absolute risk for a meaningful time period	N/A
Other analyses	17	Report other analyses done—eg analyses of subgroups and interactions, and sensitivity analyses	Results: Comparison of WHO 1997 and 2009 criteria and Biological parameters differing between DF, DHF, and DSS, and between DWWS and SD sections; Tables 3-5
**Discussion**			
Key results	18	Summarise key results with reference to study objectives	Discussion, paragraph 2, 4 and 6
Limitations	19	Discuss limitations of the study, taking into account sources of potential bias or imprecision. Discuss both direction and magnitude of any potential bias	Discussion, paragraph 3
Interpretation	20	Give a cautious overall interpretation of results considering objectives, limitations, multiplicity of	Discussion, paragraph 5, and 6 to 9

		analyses, results from similar studies, and other relevant evidence	
Generalisability	21	Discuss the generalisability (external validity) of the study results	N/A
**Other information**			
Funding	22	Give the source of funding and the role of the funders for the present study and, if applicable, for the original study on which the present article is based	Acknowledgements, paragraph 2

*Give information separately for exposed and unexposed groups.

Note: An Explanation and Elaboration article discusses each checklist item and gives methodological background and published examples of transparent reporting. The STROBE checklist is best used in conjunction with this article (freely available on the Web sites of PLoS Medicine at http://www.plosmedicine.org/, Annals of Internal Medicine at http://www.annals.org/, and Epidemiology at http://www.epidem.com/). Information on the STROBE Initiative is available at www.strobe-statement.org.
