# Supplementary material for: Comparison of dengue case classification schemes and evaluation of biological changes in different dengue clinical patterns in a longitudinal follow-up of hospitalized children in Cambodia
Source: PLoS Negl Trop Dis. 2020 Sep 14;14(9):e0008603. doi: 10.1371/journal.pntd.0008603 (PMC7515206; doi:10.1371/journal.pntd.0008603)
Supplement: S1 Appendix — (PDF) [file pntd.0008603.s002.pdf]

S1 Appendix. Description of dengue classifications used following WHO 1997 and WHO 2009 guidelines

A. WHO 1997 classification

As described in the WHO 1997 case definition, patients were classed into three groups: dengue fever, dengue hemorrhagic fever, and dengue shock syndrome.

Dengue fever (DF) is defined by fever or a history of fever lasting 2-7 days, with 2 or more of the following symptoms: headache, retro-orbital pain, myalgia, arthralgia, rash, or hemorrhagic manifestations at admission.

Dengue hemorrhagic fever (DHF) is characterized by (i) earlier and/or current presence of DF signs, (ii) hemorrhagic tendencies in terms of at least one sign (positive tourniquet test; petechiae; ecchymosis or purpura; bleeding from the mucosa, gastrointestinal tract, injection sites, or other locations; haematemesis or melaena), (iii) thrombocytopenia (≤ $100x10^9$/L), and (iv) evidence of plasma leakage due to increased vascular permeability, visible in terms of at least one of the following three cases:

1. **An increase in hematocrit greater than or equal to 20% above average for given age, sex and population**
   We used the hematocrit normal range of 32-40% for children aged from 2 to 6 years old ([2-6] y/o) and 32-49% for those aged over 6 and up to 15 years old (]6-15] y/o) (1). An increase in hematocrit level greater than or equal to 20% of the mean of the normal range for both age categories was calculated. Considering that the mean of the normal range for both age categories was 36% for [2-6] y/o and 40.5% for]6-15] y/o children, all hematocrit levels ≥ 43.2% (resp. ≥ 48.6%) at Visit 1 for children aged [2-6] y/o (resp.]6-15] y/o) were considered as an evidence of plasma leakage. We did not consider hematocrit levels at the other visits as all patients received an infusion of intravenous fluid at Visit 1.
2. **A drop in the hematocrit following volume-replacement treatment equal to or greater than 20% of baseline.**
   For all children with hematocrit levels greater than the mean values defined above for both age categories at Visit 1 or Visit 2, we looked for a 20% drop of hematocrit levels between Visit 2 and Visit 1, Visit 3 and Visit 2, and Visit 3 and Visit 1.
3. **Signs of plasma leakage such as pleural effusion, ascites, or hypoproteinemia.**
   The presence of pleural effusion, ascites, and hepatomegaly was evaluated by ultrasonographic examination performed during the three scheduled visits. However, the proteinemia level was not measured in this study.

Dengue shock syndrome (DSS) is defined as the presence of all four DHF criteria, plus evidence of circulatory failure. In this study we used weak pulse and narrow pulse pressure (defined by a difference in systolic and diastolic blood pressure of at most 20 mmHg) or hypotension with respect to age (age < 5 years: ≤ 80 mmHg; age ≥ 5 years: ≤ 90 mmHg). Additional signs such as

cold and clammy skin, and restlessness, were also considered. All of these were calculated or registered at Visits 1 and 2. Finally, note that subtle differences between DHF1/DHF2 and between DDS3/DSS4 were not taken into consideration.

B. WHO 2009 classification

Due to the difficulties in prospectively applying DHF case definition criteria for the WHO 1997 classification scheme as described earlier, a new classification scheme known as WHO 2009 was defined, separating dengue cases into severe dengue and non-severe dengue. Non-severe dengue patients are divided into two subgroups: patients with warning signs (DWWS) and those without (D-nonWS).

Dengue without warning signs (D-nonWS) is defined by fever plus at least two of the following signs: nausea, vomiting, rash, aches and pains.

Dengue with warning signs (DWWS) is defined as an association of clinical signs with laboratory findings; detection therefore requires strict observation and good medical care. Warning signs include abdominal pain or tenderness, vomiting, petechiae, purpura, bleeding nose or gum, hematemesis, and conjunctival hemorrhage, and were investigated at all visits. Clinical fluid accumulation (pleural effusion and ascites fluid) and liver enlargement were evaluated using ultrasonographic examination as previously described for DHF. We adapted WHO 2009 classification by using ultrasound that gave us the opportunity to semi-quantify the degree of fluid accumulation, providing a more accurate clinical diagnosis in discriminating between DWWS and severe dengue (SD): (i) minimal amount of liquid was considered as mild plasma leakage i.e. a warning sign (DWWS); (ii) moderate or abundant amount of liquid was considered as severe dengue, possibly leading to aggravation with respiratory distress (SD).
We also considered an increase in the hematocrit above the normal range as previously defined (i.e., 36% for [2-6] y/o and 40.5% for]6-15] y/o children) concurrent with thrombocytopenia (≤ $100x10^9$/L) as a significant marker of DWWS at Visits 1 and 2. However, all patients received an intravenous fluid infusion at the time of hospital admission, which could have contributed to normalizing or decreasing their hematocrit levels. Thus, we also considered a rapid decrease in platelet count (drop ≥ 50% between consecutive visits) as another significant marker of DWWS. Finally, an isolated thrombocytopenia (i.e., at only one visit) was not considered as a warning sign.

Severe dengue (SD) was defined by (i) severe plasma leakage leading to shock (DSS) or fluid accumulation with respiratory distress, (ii) severe bleeding as evaluated by the clinician, or (iii) severe organ involvement comprising hepatic injury (AST or ALT levels ≥ 1000 IU/L). Moreover, moderate or abundant pleural effusion detected by ultrasound was also considered a clinical sign which may lead to respiratory distress. Such patients were also classified as SD.

Reference

1. Laboratoire d'Hématologie Cellulaire du CHU d'Angers [Internet]. [cited 2019 Apr 13]. Available from: http://www.hematocell.fr/index.php/les-cellules-du-sang/15-les-cellules-du-sang-et-de-la-moelle-osseuse/valeurs-normales-de-lhemogramme-selon-lage/129-hemogramme-selon-lage
