# Supplementary material for: Comparison of dengue case classification schemes and evaluation of biological changes in different dengue clinical patterns in a longitudinal follow-up of hospitalized children in Cambodia
Source: PLoS Negl Trop Dis. 2020 Sep 14;14(9):e0008603. doi: 10.1371/journal.pntd.0008603 (PMC7515206; doi:10.1371/journal.pntd.0008603)
Supplement: S1 Table — (PDF) [file pntd.0008603.s003.pdf]

S1 Table. Interpretation criteria of virological and serological analyzes to define acute dengue infection (DI), other arbovirus infection (OAI) and non-dengue infection (NDI) groups

		Visit 1 blood sample				Visit 3 blood sample		
		Dengue RT-PCR	NS1	MAC-ELISA	HI assay	MAC-ELISA	HI assay	Conclusion
Dengue infection	n=164	Positive	Positive or Negative	Positive or Negative	Positive or Negative	Positive or Negative	Positive	**Acute dengue infection**
	n=5	Negative	Positive	Positive or Negative	Positive or Negative	Positive or Negative	Positive	**Acute dengue infection**
	n=4	Negative	Negative	Negative	Positive or Negative	Positive:<br>Anti-DENV IgM seroconversion<br>Absence of anti-JEV IgM	Positive:<br>fourfold increase in the titer of total antibodies against DENV antigen	**Acute dengue infection (seroconversion)**
Other arbovirus infections	n=12	Negative<br>Chikungunya RT-PCR positive (performed in second-line diagnosis)	Negative	Negative	Negative or Positive	Positive:<br>Anti-CHIKV IgM seroconversion	Negative or Positive with stable total antibodies titer against DENV antigen	**Acute Chikungunya infection**
	n=1	Negative<br>Chikungunya RT-PCR negative (performed in second-line diagnosis)	Negative	Negative	Negative or Positive	Positive:<br>Anti-CHIKV IgM seroconversion	Negative or Positive with stable total antibodies titer against DENV antigen	**Acute Chikungunya infection (seroconversion)**
	n=1	Positive<br>Chikungunya RT-PCR positive (performed in second-line diagnosis)	Negative	Negative	Negative or Positive	Positive:<br>Anti-DENV IgM seroconversion<br>Anti-CHIKV IgM seroconversion	Positive:<br>fourfold increase in the titer of total antibodies against DENV antigen	**Acute dengue and Chikungunya coinfection**
	n=1	Negative<br>Chikungunya RT-PCR negative (performed in second-line diagnosis)	Negative	Positive:<br>Presence of anti-CHIKV IgM	Negative or Positive	Positive:<br>Presence of anti-CHIKV IgM	Negative or Positive with stable total antibodies titer against DENV antigen	**Recent infection by Chikungunya virus**
	n=1	Negative<br>Chikungunya RT-PCR negative (performed in second-line diagnosis)	Negative	Positive:<br>Presence of anti-DENV IgM<br>Presence of anti-CHIKV IgM<br>Absence of anti-JEV IgM	Positive	Positive:<br>Presence of anti-DENV IgM Presence of anti-CHIKV IgM<br>Absence of anti-JEV IgM	Positive with stable total antibodies titer against DENV antigen	**Recent infection by dengue and Chikungunya viruses**
	n=17	Negative<br>Chikungunya RT-PCR negative (performed in second-line diagnosis)	Negative	Positive:<br>Presence of anti-DENV or anti-Flavivirus IgM	Positive	Positive:<br>Presence of anti-Flavivirus IgM	Positive with stable total antibodies titer against flavivirus antigens	**Recent flavivirus infection**
Non-Dengue	n=37	Negative	Negative	Negative	Negative or Positive	Negative	Negative or Positive with stable total antibodies titer against DENV or flavivirus antigens	**Non-dengue infection**

Abbreviation: RT-PCR: Reverse Transcription Polymerase Chain Reaction; MAC-ELISA: Immunoglobulin M Capture Enzyme-Linked Immunosorbent Assay; HI: Hemagglutination Inhibition; DENV: dengue virus: JEV: Japanese encephalitis virus; CHIKV: Chikungunya virus.
