# Supplementary figures and images for: Comparison of dengue case classification schemes and evaluation of biological changes in different dengue clinical patterns in a longitudinal follow-up of hospitalized children in Cambodia
Source: PLoS Negl Trop Dis. 2020 Sep 14;14(9):e0008603. doi: 10.1371/journal.pntd.0008603 (PMC7515206; doi:10.1371/journal.pntd.0008603)

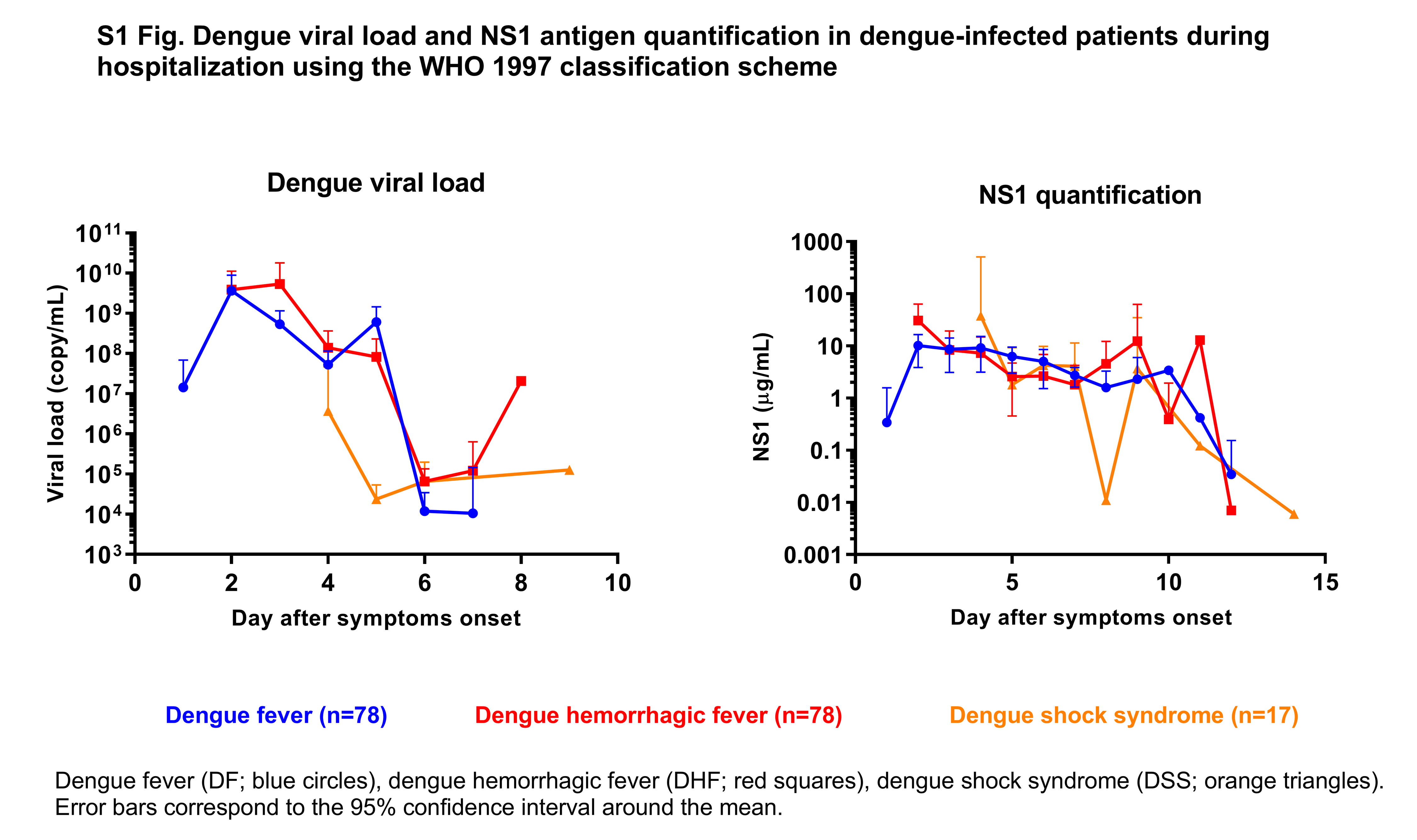

Supplement: S1 Fig — Dengue fever (DF; blue circles), dengue hemorrhagic fever (DHF; red squares), dengue shock syndrome (DSS; orange triangles). Error bars correspond to the 95% confidence interval around the mean. (TIF) [file pntd.0008603.s004.tif]

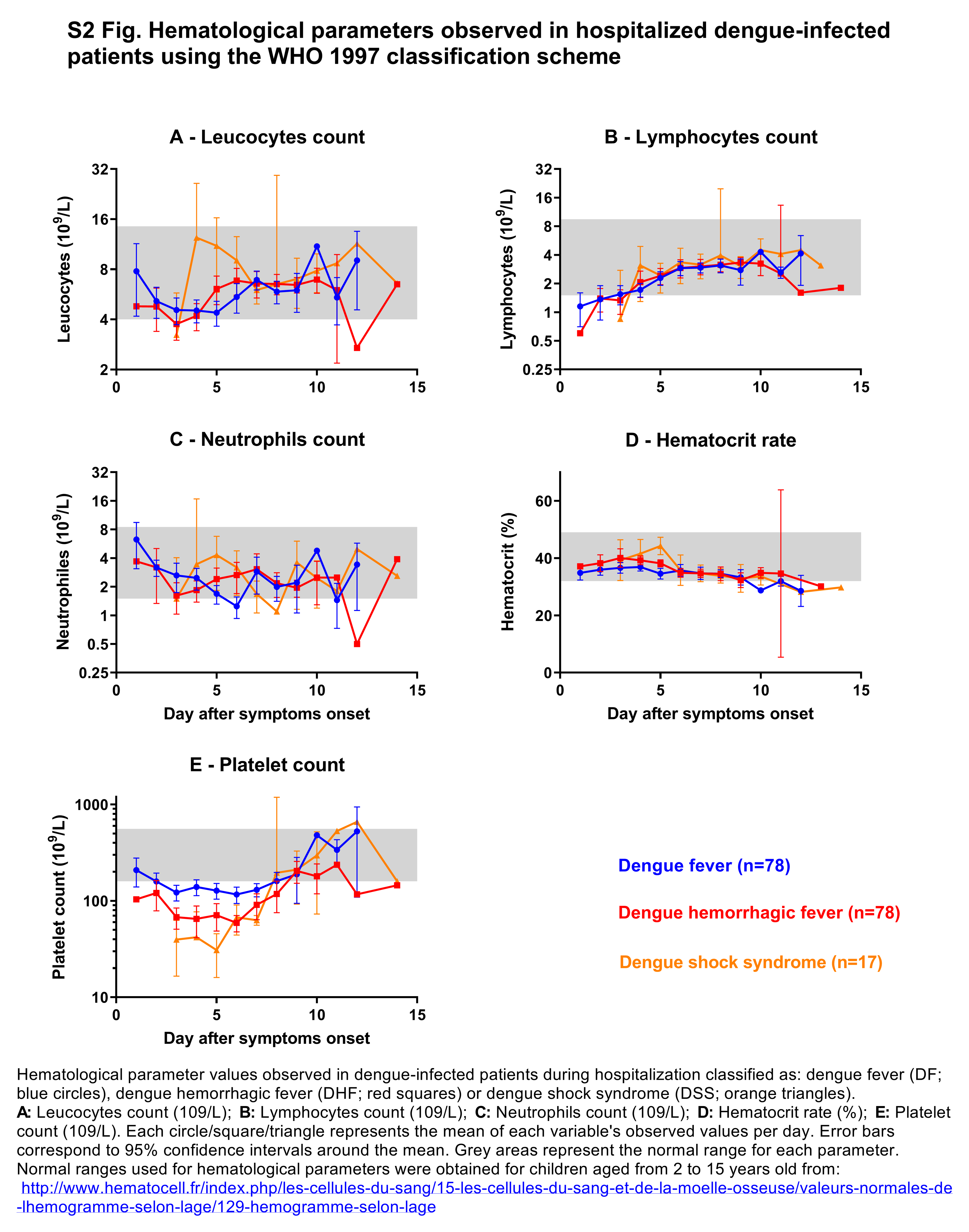

Supplement: S2 Fig — Hematological parameter values observed in dengue-infected patients during hospitalization classified as: dengue fever (DF; blue circles), dengue hemorrhagic fever (DHF; red squares) or dengue shock syndrome (DSS; orange triangles). (A) Leucocyte count (109/L); (B) Lymphocyte count (109/L); (C) Neutrophils count (109/L); (D) Hematocrit rate (%); (E) Platelet count (109/L). Each circle/square/triangle represents the mean of each variable's observed values per day. Error bars correspond to 95% confidence intervals around the mean. Grey areas represent the normal range for each parameter. Normal ranges used for hematological parameters were obtained for children aged from 2 to 15 years old from: http://www.hematocell.fr/index.php/les-cellules-du-sang/15-les-cellules-du-sang-et-de-la-moelle-osseuse/valeurs-normales-de-lhemogramme-selon-lage/129-hemogramme-selon-lage. (TIF) [file pntd.0008603.s005.tif]

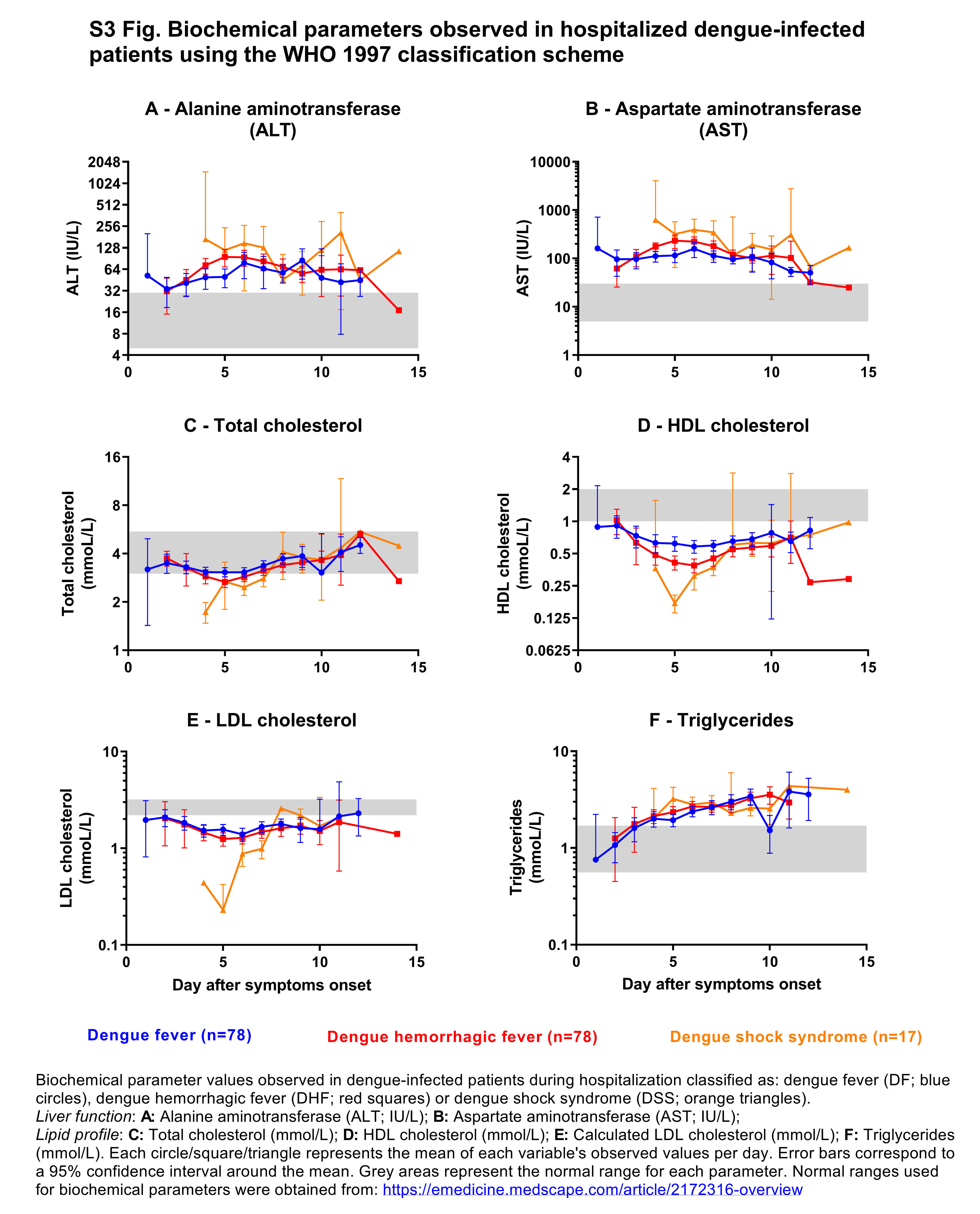

Supplement: S3 Fig — Biochemical parameter values observed in dengue-infected patients during hospitalization classified as: dengue fever (DF; blue circles), dengue hemorrhagic fever (DHF; red squares) or dengue shock syndrome (DSS; orange triangles). Liver function: (A) Alanine aminotransferase (ALT; IU/L); (B) Aspartate aminotransferase (AST; IU/L); Lipid profile: (C) Total cholesterol (mmol/L); (D) HDL cholesterol (mmol/L); (E) Calculated LDL cholesterol (mmol/L); (F) Triglycerides (mmol/L). Each circle/square/triangle represents the mean of each variable's observed values per day. Error bars correspond to a 95% confidence interval around the mean. Grey areas represent the normal range for each parameter. Normal ranges used for biochemical parameters were obtained from: https://emedicine.medscape.com/article/2172316-overview. (TIF) [file pntd.0008603.s006.tif]

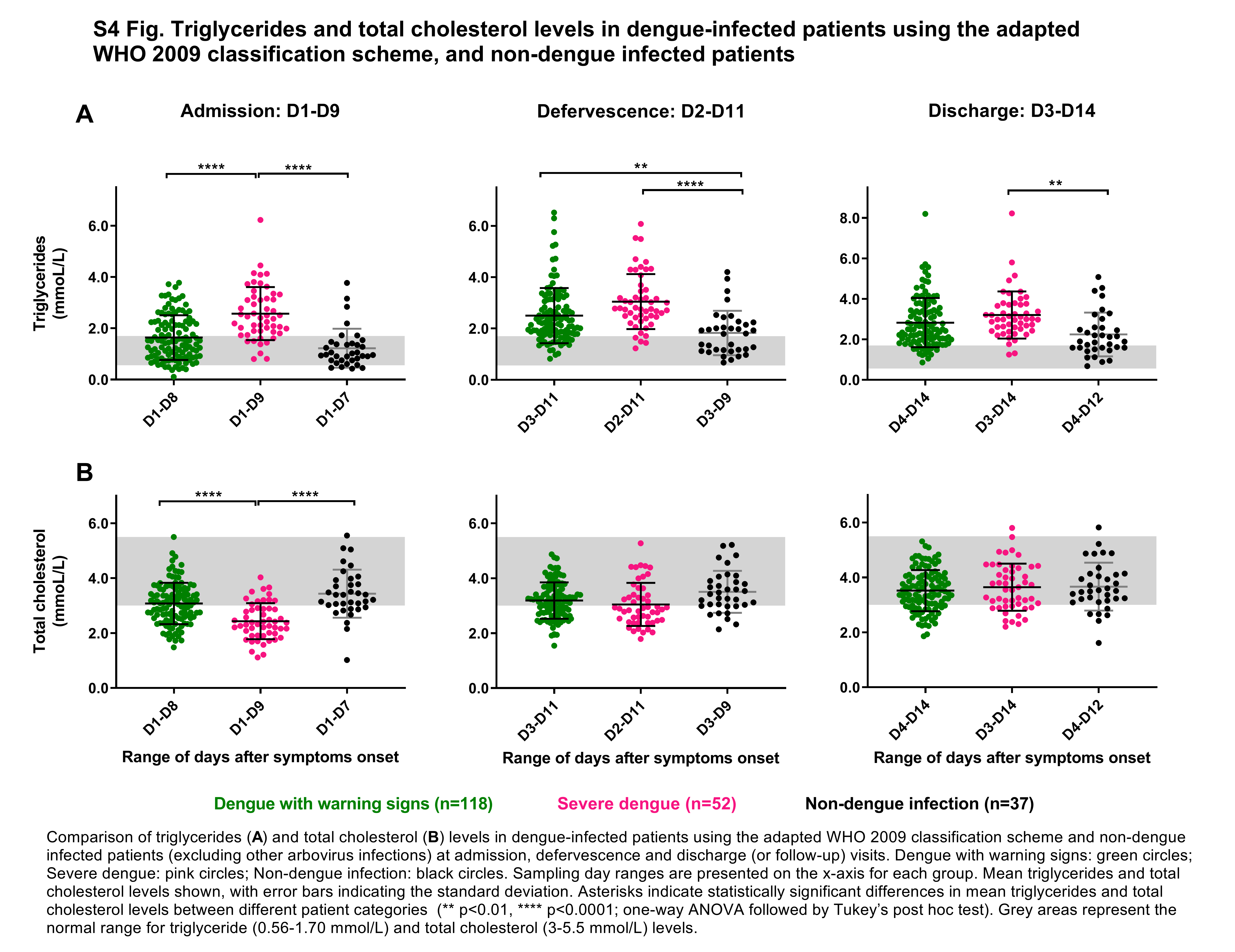

Supplement: S4 Fig — Comparison of triglyceride (A) and total cholesterol (B) levels in dengue-infected patients using the adapted WHO 2009 classification scheme and non-dengue infected patients (excluding other arbovirus infections) at admission, defervescence and discharge (or follow-up) visits. Dengue with warning signs: green circles; Severe dengue: pink circles; Non-dengue infection: black circles. Sampling day ranges are presented on the x-axis for each group. Mean triglycerides and total cholesterol levels shown, with error bars indicating the standard deviation. Asterisks indicate statistically significant differences in mean triglycerides and total cholesterol levels between different patient categories (** p<0.01, **** p<0.0001; one-way ANOVA followed by Tukey’s post hoc test). Grey areas represent the normal range for triglyceride (0.56–1.70 mmol/L) and total cholesterol (3–5.5 mmol/L) levels. (TIF) [file pntd.0008603.s007.tif]

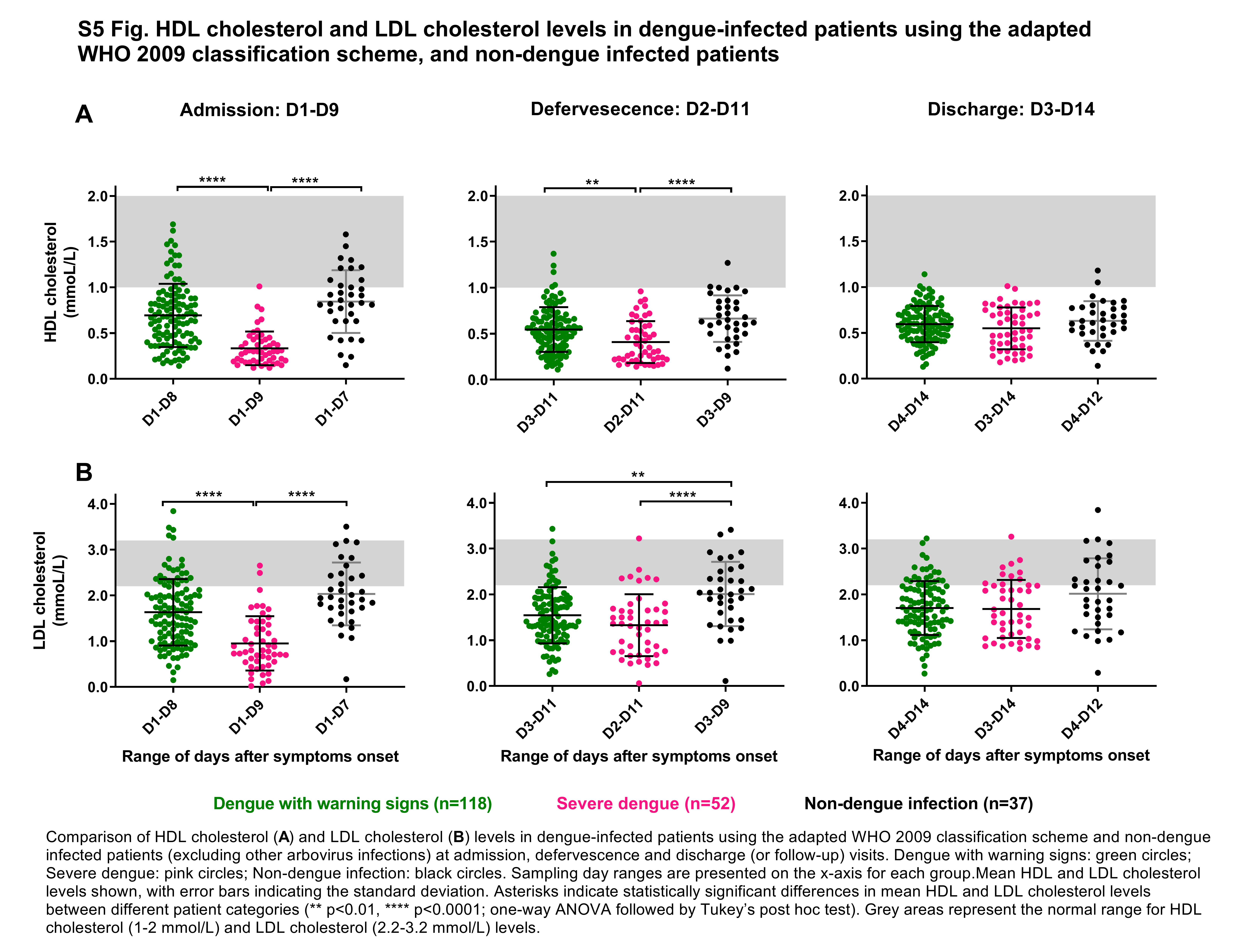

Supplement: S5 Fig — Comparison of HDL cholesterol (A) and LDL cholesterol (B) levels in dengue-infected patients using the adapted WHO 2009 classification scheme and non-dengue infected patients (excluding other arbovirus infections) at admission, defervescence and discharge (or follow-up) visits. Dengue with warning signs: green circles; Severe dengue: pink circles; Non-dengue infection: black circles. Sampling day ranges are presented on the x-axis for each group. Mean HDL and LDL cholesterol levels shown, with error bars indicating the standard deviation. Asterisks indicate statistically significant differences in mean HDL and LDL cholesterol levels between different patient categories (** p<0.01, **** p<0.0001; one-way ANOVA followed by Tukey’s post hoc test). Grey areas represent the normal range for HDL cholesterol (1–2 mmol/L) and LDL cholesterol (2.2–3.2 mmol/L) levels. (TIF) [file pntd.0008603.s008.tif]

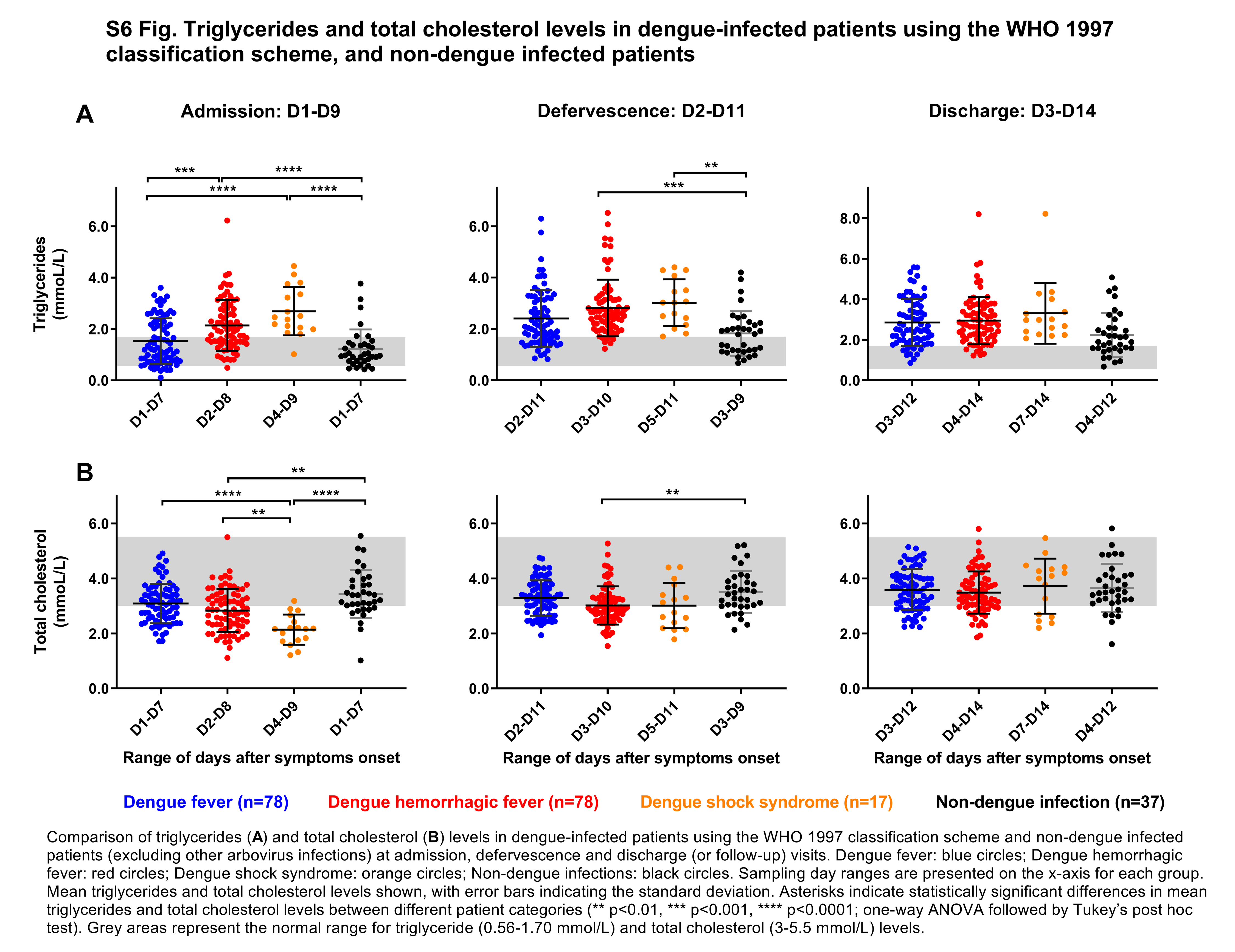

Supplement: S6 Fig — Comparison of triglycerides (A) and total cholesterol (B) levels in dengue-infected patients using the WHO 1997 classification scheme and non-dengue infected patients (excluding other arbovirus infections) at admission, defervescence and discharge (or follow-up) visits. Dengue fever: blue circles; Dengue hemorrhagic fever: red circles; Dengue shock syndrome: orange circles; Non-dengue infections: black circles. Sampling day ranges are presented on the x-axis for each group. Mean triglycerides and total cholesterol levels shown, with error bars indicating the standard deviation. Asterisks indicate statistically significant differences in mean triglycerides and total cholesterol levels between different patient categories (** p<0.01, *** p<0.001, **** p<0.0001; one-way ANOVA followed by Tukey’s post hoc test). Grey areas represent the normal range for triglyceride (0.56–1.70 mmol/L) and total cholesterol (3–5.5 mmol/L) levels. (TIF) [file pntd.0008603.s009.tif]

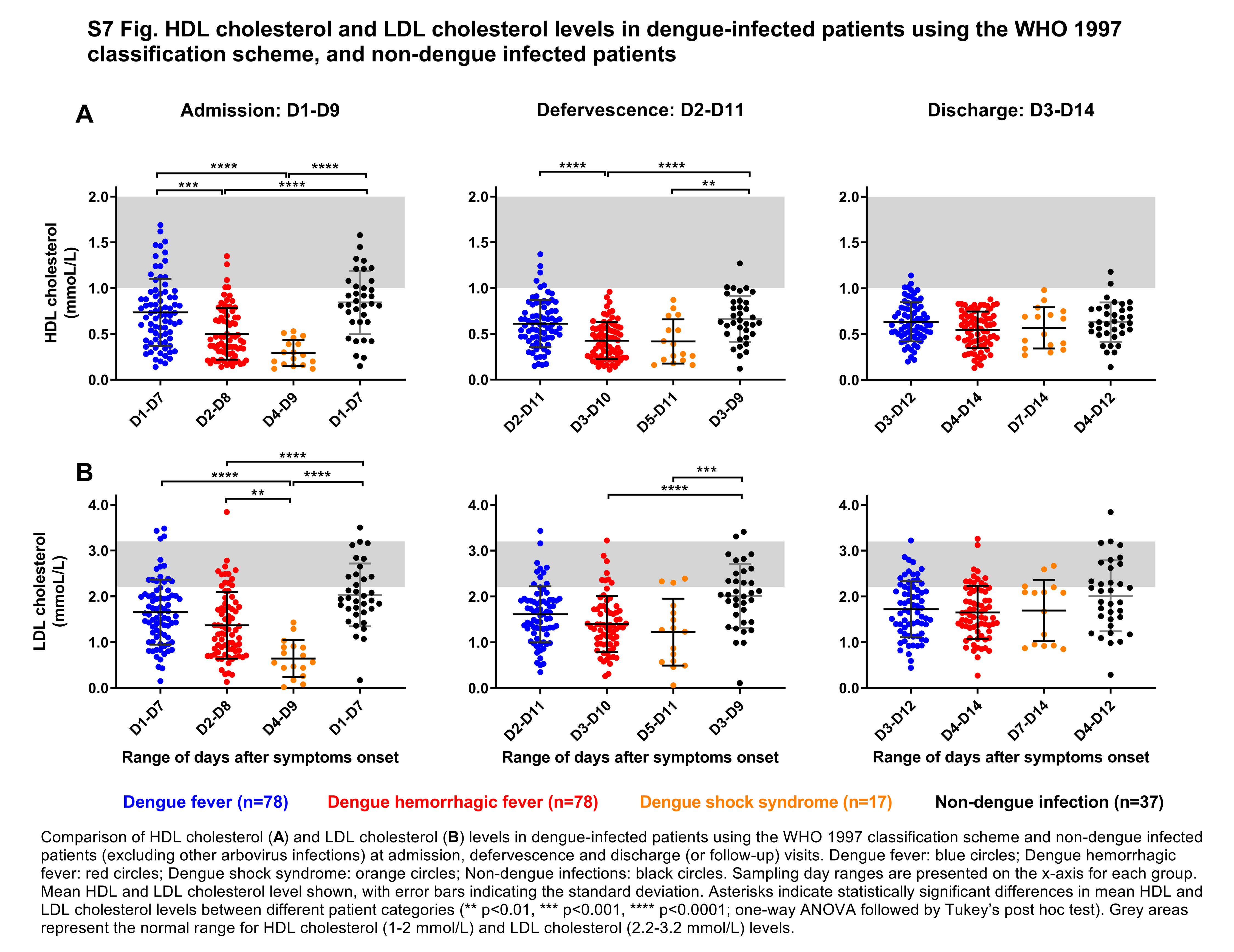

Supplement: S7 Fig — Comparison of HDL cholesterol (A) and HDL cholesterol (B) levels in dengue-infected patients using the WHO 1997 classification scheme and non-dengue infected patients (excluding other arbovirus infections) at admission, defervescence and discharge (or follow-up) visits. Dengue fever: blue circles; Dengue hemorrhagic fever: red circles; Dengue shock syndrome: orange circles; Non-dengue infections: black circles. Sampling day ranges are presented on the x-axis for each group. Mean HDL and LDL cholesterol level shown, with error bars indicating the standard deviation. Asterisks indicate statistically significant differences in mean HDL and LDL cholesterol levels between different patient categories (** p<0.01, *** p<0.001, **** p<0.0001; one-way ANOVA followed by Tukey’s post hoc test). Grey areas represent the normal range for HDL cholesterol (1–2 mmol/L) and LDL cholesterol (2.2–3.2 mmol/L) levels. (TIF) [file pntd.0008603.s010.tif]
